# Kinetics of the thapsigargin-induced Ca^2+^ mobilisation: A quantitative analysis in the HEK-293 cell line

**DOI:** 10.3389/fphys.2023.1127545

**Published:** 2023-03-27

**Authors:** Tillman Pick, Igor Gamayun, René Tinschert, Adolfo Cavalié

**Affiliations:** Experimental and Clinical Pharmacology and Toxicology, Pre-clinical Center for Molecular Signalling (PZMS), Saarland University, Homburg, Germany

**Keywords:** Ca^2+^ homeostasis, Ca^2+^ leak, store-operated Ca^2+^ entry, Ca^2+^ imaging, thapsigargin

## Abstract

Thapsigargin (TG) inhibits the sarco/endoplasmic reticulum Ca^2+^ ATPase (SERCA) pump and, when applied acutely, it initiates a Ca^2+^ mobilisation that begins with the loss of Ca^2+^ from the endoplasmic reticulum (ER) and culminates with store-operated Ca^2+^ entry (SOCE) from the extracellular space. Using the popular model cell line HEK-293, we quantified TG-induced changes in cytosolic and ER Ca^2+^ levels using FURA-2 and the FRET-based ER Ca^2+^ sensor D1ER, respectively. Our analysis predicts an ER Ca^2+^ leak of 5–6 µM⋅s^−1^ for the typical basal ER Ca^2+^ level of 335–407 µM in HEK-293 cells. The resulting cytosolic Ca^2+^ transients reached peak amplitudes of 0.6–1.0 µM in the absence of external Ca^2+^ and were amplified by SOCE that amounted to 28–30 nM⋅s^−1^ in 1 mM external Ca^2+^. Additionally, cytosolic Ca^2+^ transients were shaped by a Ca^2+^ clearance of 10–13 nM⋅s^−1^. Using puromycin (PURO), which enhances the ER Ca^2+^ leak, we show that TG-induced cytosolic Ca^2+^ transients are directly related to ER Ca^2+^ levels and to the ER Ca^2+^ leak. A one-compartment model incorporating ER Ca^2+^ leak and cytosolic Ca^2+^ clearance accounted satisfactorily for the basic features of TG-induced Ca^2+^ transients and underpinned the rule that an increase in amplitude associated with shortening of TG-induced cytosolic Ca^2+^ transients most likely reflects an increase in ER Ca^2+^ leak.

## 1 Introduction

Non-excitable cells generate cytosolic Ca^2+^ signals as a response to the stimulation of G-protein-coupled receptors and growth factor receptors (Berridge et al., 2003; Clapham, 2007). Typically, these Ca^2+^ signals can be observed for a short period of time in the absence of external Ca^2+^, indicating that the primary mechanism for the underlying increase in the cytosolic Ca^2+^ concentration ([Ca^2+^]_cyt_) is the release of Ca^2+^ from the endoplasmic reticulum (ER) (Barak and Parekh, 2020). Along with mitochondria, the clearance of cytosolic Ca^2+^ by the plasma membrane Ca^2+^ ATPase (PMCA) and the Na^+^-Ca^2+^-exchanger (NCX) reduces the amount of Ca^2+^ that is available to the sarco/endoplasmic reticulum Ca^2+^ ATPase (SERCA) for refilling ER Ca^2+^ stores after each Ca^2+^ spike, and as a consequence, Ca^2+^ signals run down after a few minutes in Ca^2+^-free solutions (Barak and Parekh, 2020). To generate stable high cytosolic Ca^2+^ spikes, the influx of extracellular Ca^2+^ is therefore obligatory, and this is achieved by the store-operated Ca^2+^ entry (SOCE), so called because it is triggered by stimuli that reduce ER Ca^2+^ levels (Putney, 2017; Lewis, 2020). In general, SOCE generates high cytosolic Ca^2+^ levels necessary to refill ER Ca^2+^ stores, meaning that the homeostatic control of ER Ca^2+^ is essential for cells to be able to generate cytosolic Ca^2+^ signals.

Early studies of the ER counterpart of muscle cells, the sarcoplasmic reticulum (SR), revealed that this organelle stores Ca^2+^ in an ATP-dependent manner (Hasselbach, 1966). Now, it is widely accepted that the ER/SR represents the major Ca^2+^ reservoir in the cell, whereby SERCA pumps transport Ca^2+^ from cytosol into the ER lumen, where it is buffered by chaperones such as calreticulin, calnexin and the binding-immunoglobulin protein (BiP) (Vandecaetsbeek et al., 2011; Wang et al., 2019). Notably, the ER membrane seems to have a finite permeability for small molecules and Ca^2+^, which gives rise to a Ca^2+^ leak from the ER (Camello et al., 2002; Lemos et al., 2021). This form of ER Ca^2+^ leak appears to be different from the SR Ca^2+^ leak, which is understood as a Ca^2+^ release other than during the E-C coupling process in muscle cells and is believed to be supported by ryanodine receptors (Bers, 2014). So far, it seems that various parallel pathways account for the ER Ca^2+^ leak. Several studies have identified the Sec61 translocon as a Ca^2+^ leak channel in the ER, which is tightly controlled by BiP and Ca^2+^-calmodulin (Lang et al., 2017). Other ion channels such as ORAI3, TRPC1, and BI-1 have been also reported to mediate Ca^2+^ leak from the ER, aside from the evoked Ca^2+^ release through IP_3_ and ryanodine receptors (Camello et al., 2002; Lemos et al., 2021; Parys and van Coppenolle, 2022). Therefore, it is not surprising that thapsigargin (TG) became a popular substance used experimentally to induce ER Ca^2+^ depletion. TG is a potent inhibitor of SERCA pumps, which prevents the refilling of ER Ca^2+^ stores with the consequence that the ER Ca^2+^ concentration ([Ca^2+^]_ER_) decreases after the exposure of cells to TG (Thastrup et al., 1990; Treiman et al., 1998). When TG is used to inhibit SERCA pumps in the absence of extracellular Ca^2+^, the transient increase in [Ca^2+^]_cyt_ is assumed to reflect the Ca^2+^ leak from the ER (Prakriya and Lewis, 2015; Lang et al., 2017), as removal of external Ca^2+^ ensures that the cytosolic TG-triggered Ca^2+^ accumulation is not contaminated by Ca^2+^ fluxes other than those from the ER. Such Ca^2+^ accumulation in the cytosol is efficiently reduced by clearance across the plasma membrane, so that subsequent re-addition of external Ca^2+^ prompts again an accumulation of cytosolic Ca^2+^, which in this case reflects mainly SOCE (Prakriya and Lewis, 2015). Thus, TG and other SERCA inhibitors such as cyclopiazonic acid (CPA) and 2,5-di (tert-butyl)hydroquinone (DBHQ) are crucial in elucidating mechanism of intracellular Ca^2+^ signalling (Tadini-Buoninsegni et al., 2018).

While the introduction of FURA-2 to image [Ca^2+^]_cyt_ combined with TG to deplete ER Ca^2+^ made feasible the detection of SOCE in numerous cell types, electrophysiological techniques used to measure Ca^2+^ currents defined the prototypical store-operated channels, i.e., the Ca^2+^-release-activated Ca^2+^ (CRAC) channels that are formed by ORAI proteins in the plasma membrane (Parekh and Penner, 1997; Prakriya and Lewis, 2015). In contrast, electrophysiological studies of the Ca^2+^ leak from the ER are hampered because ER membranes are not directly accessible to ion current recordings. Only the incorporation of Sec61 translocons in artificial membranes has demonstrated that these proteins are able to function as Ca^2+^-permeable ion channels (Simon and Blobel, 1991; Wirth et al., 2003). Therefore, live cell Ca^2+^ imaging remains an essential tool in studies of the Ca^2+^ leak from the ER. For instance, this imaging technique combined with siRNA-mediated gene silencing has been used to identify Ca^2+^ leak channels in the ER membrane and their regulatory mechanisms (Lang et al., 2011a). Specifically, combined imaging of cytosolic and ER/SR Ca^2+^ provided compelling information on the mechanisms of the intracellular Ca^2+^ dynamics in various cell types (Solovyova et al., 2002; Zima et al., 2010; Rojo-Ruiz et al., 2021; Blum and Schulte, 2022; Dagnino-Acosta and Guerrero-Hernández, 2022). In the present study, we compile data obtained in our laboratory on the Ca^2+^ mobilisation induced by TG in the model cell line HEK-293. We used FURA-2 either separately or simultaneously with the FRET based Ca^2+^ sensor D1ER to image [Ca^2+^]_cyt_ and [Ca^2+^]_ER_, respectively (Gamayun et al., 2019; Pick et al., 2021). The Ca^2+^ leak from ER was modulated with puromycin (PURO), which is a structural analogue of t-RNA and, as such, a substrate of the peptidyltransferase of the 60 S subunit of ribosomes. By its incorporation in the nascent peptides, PURO causes the release of incomplete peptides and enhances the Ca^2+^ leak from ER by promoting a Ca^2+^-permeable state of Sec61 translocons (Simon and Blobel, 1991; Van Coppenolle et al., 2004; Lang et al., 2011b). Using this approach, we quantified the contribution of the ER Ca^2+^ leak to the generation of cytosolic Ca^2+^ transients and generated a model for the TG-induced Ca^2+^ mobilisation that comprises quantitative data on the Ca^2+^ leak from the ER and the clearance of cytosolic Ca^2+^ in HEK-293 cells.

## 2 Materials and methods

### 2.1 Cell culture

HEK-293 cells (ATCC CRL-1573) were cultivated in Minimal Essential Medium (MEM) supplemented with 10% (v/v) foetal bovine serum (FBS). We generated the cell line HEK-D1ER that stably expresses the FRET-based D1ER sensor in the ER lumen (Gamayun et al., 2019). D1ER was kindly provided by R. Y. Tsien (University of California San Diego, La Jolla, United States). HEK-D1ER cells were maintained in culture under selection with G418 (0.5 mg/mL) in MEM supplemented with 10% (v/v) FBS. Cell culture was carried out at 37°C in a humidified environment with 5% CO_2_.

### 2.2 Reagents and recording solutions

Thapsigargin and ionomycin (TG, IONO; Thermo Fisher Scientific) were dissolved in DMSO to obtain 1 mM and 10 mM stocks, respectively. TG and IONO stock solutions were maintained at −20°C in the dark and dilutions were made directly in the recording solution just before experiments. TG and IONO were applied “online” to the cells while the Ca^2+^ imaging was running. In order to avoid problems arising from slow mixing, we added 2× solutions of these substances to the bath at a ratio of 1:1. Typically, the end concentration of TG and IONO were 1 and 10 μM, respectively. The final DMSO concentration in the recording chamber was maximally 0.1% (v/v). Puromycin dihydrochloride (PURO; Sigma-Aldrich) was dissolved in a Ca^2+^-containing solution to obtain 10 mM stock and dissolved further as required. Just before Ca^2+^ imaging experiments, cells were incubated for 10 min in a 2 mM Ca^2+^ solution (140 mM NaCl, 4 mM KCl, 1 mM MgCl_2_, 2 mM CaCl_2_, 10 mM glucose, 10 mM HEPES-KOH, pH 7.3) that contained various PURO concentrations.

FURA-2 AM (Thermo Fisher Scientific) was dissolved in DMSO to obtain a 1 mM stock solution. Cells were loaded with FURA-2 prior to imaging experiments by incubation with a 1 mM Ca^2+^ solution containing 4 µM FURA-2 AM for 20 min, washed and subsequently exposed to the recording solution.

The initial Ca^2+^ imaging experiments were performed in the presence of external Ca^2+^ with recording solutions containing 1 or 10 mM Ca^2+^ (140 mM NaCl, 4 mM KCl, 1 mM MgCl_2_, 1 or 10 mM CaCl_2_, 10 mM glucose, 10 mM HEPES-KOH, pH 7.3–7.4). Subsequently, a Ca^2+^-free recording solution (140 mM NaCl, 4 mM KCl, 1 mM MgCl_2_, 0.5 mM EGTA, 10 mM glucose and 10 mM HEPES-KOH, pH 7.3–7.4) was used in most experiments to abolish the Ca^2+^ entry from the extracellular space. In Ca^2+^ re-addition experiments, TG was applied to cells bathed in a nominal Ca^2+^-free solution (140 mM NaCl, 4 mM KCl, 1 mM MgCl_2_, 10 mM glucose and 10 mM HEPES-KOH, pH 7.3–7.4) and subsequently, Ca^2+^-containing solutions were added to obtain 1 and 10 mM free Ca^2+^ after dilution in the recording chamber. The nominal Ca^2+^-free solution was also used in Ca^2+^ clearance experiments, in which cells were first exposed to TG for 8 min in this solution. The Ca^2+^ imaging was then started and a Ca^2+^-containing solution was added to raise the Ca^2+^ concentration in the recording chamber to 0.5 mM free Ca^2+^. Subsequently, Ca^2+^ was chelated in the recording chamber by adding further a EGTA-containing solution to attain a final concentration of 2 mM EGTA after dilution in the recording chamber.

### 2.3 Live cell calcium imaging

The cytosolic Ca^2+^ ([Ca^2+^]_cyt_) was imaged with FURA-2 as previously described (Lang et al., 2011a; Pick et al., 2021). FURA-2 was excited at 340 and 380 nm alternately. The emitted fluorescence light was captured at 510 nm to obtain FURA-2 images at 340 and 380 nm excitation. FURA-2 image pairs containing 30–50 cells/frame were obtained every 3 s at a magnification of 20×. FURA-2 signals were quantified in FURA-2 image pairs as F_340_/F_380_, where F_340_ and F_380_ correspond to the background-subtracted fluorescence intensity at 340 and 380 nm excitation wavelengths, respectively. [Ca^2+^]_cyt_ was calculated with the standard ratiometric equation [Ca^2+^]_cyt_ = K_FURA-2_ ⋅ (R - R_min_)/(R_max_ - R), in which R = F_340_/F_380_ and K_FURA-2_ represents the system specific apparent Ca^2+^ dissociation constant for FURA-2 (Grynkiewicz et al., 1985; Pick et al., 2021). FURA-2 signals are given as [Ca^2+^]_cyt_.

As previously described, ER and cytosolic Ca^2+^ concentrations ([Ca^2+^]_ER_, [Ca^2+^]_cyt_) were imaged simultaneously using the FRET-based D1ER sensor and FURA-2, respectively (Gamayun et al., 2019). Firstly, HEK-D1ER cells that stably express D1ER in the ER lumen were exposed to 433 nm and the emitted fluorescence light was split at 469/23 nm and 536/27 nm to obtain the CFP and citrine components, respectively (Palmer et al., 2004). The cell fluorescence light was additionally passed through a dichrotome and projected on the chip of the microscope camera to obtain simultaneously CFP and citrine images. Secondly, FURA-2 was excited by alternated excitation at 340 and 380 nm. The emitted fluorescence light was also passed through the dichrotome and captured at 510 nm to obtain FURA-2 images at 340 and 380 nm excitation. D1ER and FURA-2 image pairs containing 5–10 cells/frame were obtained at 60× magnification every 10 s. The FRET ratios were calculated from background-subtracted CFP and citrine image pairs as F_Citrine_/F_CFP_, where F_Citrine_ and F_CFP_ represent the citrine and CFP fluorescence intensities, respectively. [Ca^2+^]_ER_ was calculated with the standard ratiometric equation [Ca^2+^]_ER_ = K_D1ER_ ⋅ (R - R_min_)/(R_max_ - R), in which R = F_Citrine_/F_CFP_ and K_D1ER_ represents the system specific apparent Ca^2+^ dissociation constant for D1ER (Palmer et al., 2004; Gamayun et al., 2019). FURA-2 signals were quantified in the FURA-2 image pairs as F_340_/F_380_, which was used to calculate [Ca^2+^]_cyt_, as previously described (Gamayun et al., 2019; Pick et al., 2021).

### 2.4 LSM microscopy

For visualization of the plasma membrane and the ER, HEK-293 cells were incubated with Cell Mask Green Plasma Membrane Stain (Thermo Fisher) with a dilution of 1:2000 and 1 µM ER-Tracker Red (BODIPY TR Glibenclamide) (Thermo Fisher), respectively, for 20 min at 37°C in HBSS containing Mg^2+^ and Ca^2+^. Images were acquired on a Zeiss LSM 880 (Zeiss, Germany) with a 63× oil objective (NA 1.4, Plan Apochromat) and 488 nm, 543 nm excitation light using Zen (Zeiss, Germany) software. Z-stack images were acquired every 500 nm throughout the entire cell to determine the cell, ER and nucleus area and the total RFP fluorescence. Subsequently, images were analyzed and processed with ImageJ (version 1.53t). Before analyzing the images, the background was subtracted using the rolling ball plugin with a radius of 50 pixels. To measure the area of the ER, a threshold was set at 20 times the mean background intensity, so that the outlines of the cells were not visible, allowing the ER area to be quantified.

### 2.5 Statistics

Single cell data has been obtained in independent Ca^2+^ imaging recordings with 3–12 coverslips per experimental setting. Because of technical reasons, we imaged only 10–15 cells per coverslip in experiments with D1ER and FURA-2, while 30–50 cells per coverslip were imaged with FURA-2 alone. The total number of analysed cells in each experimental setting is given in the figure legends. Data is given as mean ± SEM. Statistical significance (*p*-values) was calculated using the non-parametric two sample Kolmogorov-Smirnov test.

## 3 Results

HEK-293 cells are derived from embryonic human kidney tissue and exhibit an epithelial morphology. Together with HeLa cells, the HEK-293 cells are the most popular cell lines in basic biomedical research as well as in industrial biotechnology and toxicology research. HEK-293 cells are adherent, have a short doubling time of about 36 h and can be used easily for transient and stable transfection. All these advantages are specially appreciated in studies of ion channels, intracellular signalling and Ca^2+^ homeostasis (Zhang et al., 2022). In the present report, we present a data compilation of the basic parameters of Ca^2+^ dynamics for the HEK-293 cell line, as a paradigm in Ca^2+^ imaging experiments.

### 3.1 The ER size in HEK-293 cells

**FIGURE 1:**
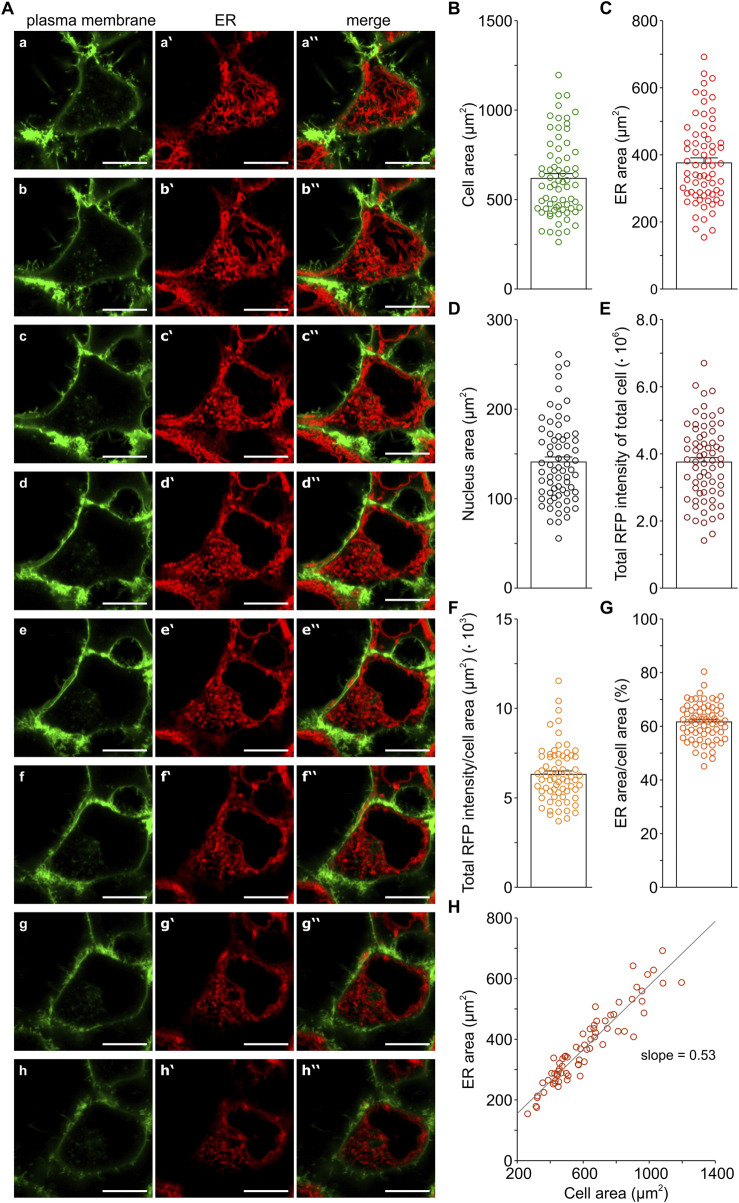
Morphology of the ER in HEK-293 cells. The plasma membrane (green) was stained with an GFP-based marker (Cell Mask Plasma Membrane Stain) and the ER (red) with an RFP-based ER tracker (Bodipy TR Glibenclamide), as described in Methods. Representative z-stack images show the plasma membrane **(A–H)** and ER staining (**A**, **a‵**-**h‵**) as well as the corresponding merged images (**A**, **a‶-h‶**) from a HEK-293 cell. Images of the z-stack are lined up from the lowermost (**a**, **a‵**, **a‶**) to the uppermost (**h**, **h‵**, **h‶**) z-plane with respect to the coverslip. Scales represent 10 µm. The areas occupied by the cell, ER, and nucleus were determined for individual HEK-293 cells in the z-stacks **(B–D)**. The amount of ER staining was measured as the absolute and normalised total RFP intensity per cell **(E,F)**. The fraction of the cytosol occupied by ER structures was calculated as ER area/cell area **(G)**. The plot of ER area vs. cell area shows a linear correlation with a slope of 0.53 and a correlation of *r* ^2^ = 0.88. Bar charts represent mean ± SEM; symbols show single cell data. Number of cells: 69.

Figure 1 illustrates the ER morphology in HEK-293 cells that we analysed using LSM microscopy. The cell membrane was stained with a GFP-based marker (Figure 1A, a-h) and the ER was visualised with an RFP-based marker (Figure 1A, a‵-h‵). As expected, HEK-293 cells displayed the typical flat morphology of adherent cells. For the quantitative analysis of the z-stacks, we used the cell membrane and ER staining to delimit the areas occupied by the cell and ER, respectively, while nuclei areas were defined as the non-stained enclosures within ER structures. In this way, we quantified the areas occupied by the cell as well as the ER and nuclei areas (Figures 1B–D). This analysis was performed with a homogeneously stained cell population, as it was substantiated by measurements of RFP intensities that showed an evenly ER staining in HEK-293 cells (Figures 1E, F). All in all, it appears that ER areas are larger than those of the nuclei. Furthermore, our quantification of ER areas suggested the presence of a prominent ER in HEK-293 cells, as illustrated by the merged images, where ER staining covers a considerable portion of the cytoplasm throughout all z-planes in the stack (Figure 1A, a‶-h‶). So far, little quantitative information is available on mammalian cell dimensions, but it is generally believed that the ER lumen generally occupies more than 10% of the total cell volume (e.g., Alberts et al., 2002). Hence, our measurements of cross-section areas strongly suggested that the ER of HEK-293 cells may be larger than previously assumed, although an optimal correlation between volumes and cross-sections in z-stacks will require a 3D reconstruction of the images.

Based on the measurements of cell and ER areas (Figures 1B, C), we calculated the ratio of ER to cell area and found that ER structures occupied approx. 61% of the cell cross-sections in the z-stacks of HEK-293 cells (Figure 1G). Furthermore, we observed a linear correlation between ER and cell area with a slope of 0.53 (Figure 1H). Thus, the average ratio of ER to cell area and the slope of the correlation between ER and cell area indicate that the ER area fraction of HEK-293 cells is in the range of 0.53–0.61. To obtain a rough estimate of the ER volume fraction in HEK-293 cells, we used for simplicity the following equation: volume fraction = (area fraction) ^3/2^ (Bakunts et al., 2017). Following this calculation, our results indicate that the ER likely occupies in the range of 38%–48% of the volume of HEK-293 cells, supporting the suggestion that HEK-293 cells possess a prominent ER.

### 3.2 The TG-induced Ca^2+^ mobilisation in HEK-293 cells

**FIGURE 2:**
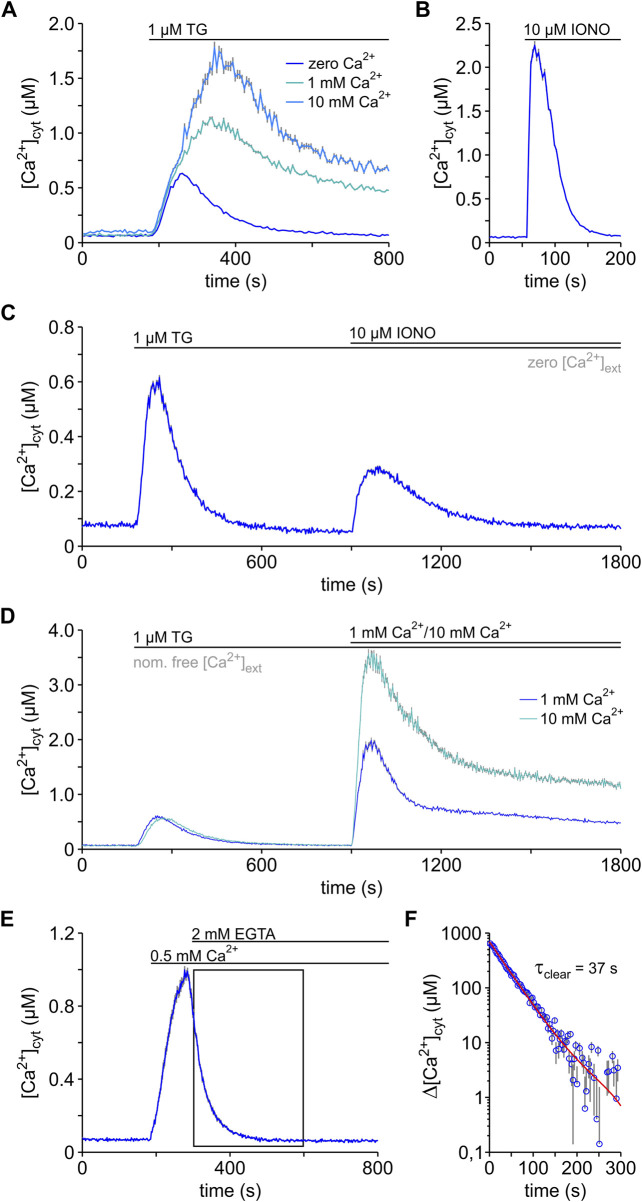
Dynamics of the Ca^2+^ mobilisation in HEK-293 cells. Changes in the cytosolic Ca^2+^ concentration ([Ca^2+^]_cyt_) were imaged using FURA-2. **(A)** Ca^2+^ mobilisation was induced by applying thapsigargin (1 μM TG) “online” to HEK-293 cells exposed to bath solutions containing either zero (0.5 mM EGTA), 1 or 10 mM free Ca^2+^ (1 mM Ca^2+^; 10 mM Ca^2+^). **(B)** The content of total Ca^2+^ in the cells was assessed with ionomycin (10 µM IONO) in the absence of external Ca^2+^, to prevent entry of external Ca^2+^. **(C)** The content of ER and non-ER Ca^2+^ was estimated by applying thapsigargin (1 μM TG) and ionomycin (10 µM IONO) sequentially to HEK-293 cells in the absence of external Ca^2+^ (zero [Ca^2+^]_ext_). **(D)** The store-operated Ca^2+^ entry (SOCE) was analysed using the so-called Ca^2+^re-addition protocol, in which the cells were exposed to TG in a bath solution with nominally free Ca^2+^ (nom. free [Ca^2+^]_ext_) and SOCE was initiated by raising external Ca^2+^ to 1 or 10 mM (1 mM Ca^2+^; 10 mM Ca^2+^). **(E,F)** The clearance of cytosolic Ca^2+^ was measured following an 8 min TG exposure in a nominal Ca^2+^-free solution (not shown). High cytosolic Ca^2+^ levels were achieved by raising external Ca^2+^ to 0.5 mM (0.5 mM Ca^2+^) and the process of cytosolic Ca^2+^ clearance was unmasked by chelating the external Ca^2+^ with EGTA (2 mM EGTA) **(E)**. The section marked with a box in E was fitted with an exponential function (red line) that has a time constant (τ_clear_) of 37 s using Eq. 3 **(F)**. Protocols of TG and IONO application and changes of [Ca^2+^]_ext_ are depicted above the graphs. Data is given as mean ± SEM. Number of cells: 37–472.

The signalling of G-protein-coupled receptors to intracellular effector proteins comprises various pathways, which in many cases involve the mobilisation of intracellular Ca^2+^ (Dhyani et al., 2020). Experimentally, Ca^2+^ mobilisation has been induced with TG in multiple cell types (Zima et al., 2010; Erdmann et al., 2011; Ikeya et al., 2014; Ferdek et al., 2017; Merino-Wong et al., 2021). Hence, we first tested the effects of 1 μM TG in HEK-293 cells in the absence and presence of external Ca^2+^ (Figure 2A). Cytosolic Ca^2+^ was imaged with FURA-2. As in other cell lines, the basal [Ca^2+^]_cyt_ (b[Ca^2+^]_cyt_) increased in HEK-293 cells depending on the external Ca^2+^ concentration ([Ca^2+^]_ext_), but it generally remained below 100 nM (Table 1). Shortly after TG application, a surge of cytosolic Ca^2+^ was observed independently of the presence of external Ca^2+^, indicating that the increase of [Ca^2+^]_cyt_ requires the release of Ca^2+^ from intracellular stores into the cytosol. Since TG is selective for SERCA pumps, it is generally accepted that TG unmasks specifically the Ca^2+^ leak from ER. Hence, the TG-induced surge of cytosolic Ca^2+^ reflects mainly the ER Ca^2+^ leak in the absence of external Ca^2+^. When Ca^2+^ was present in the external solution, the cytosolic Ca^2+^ transients were much higher than those in external free Ca^2+^ solutions, indicating that Ca^2+^ ions flowing from the extracellular space also contribute to the surges in [Ca^2+^]_cyt_ observed after TG application. This Ca^2+^ influx corresponds to SOCE, because it is activated upon depletion of internal stores (Putney, 2017; Lewis, 2020).

**TABLE 1:** Characteristic parameters of the TG-induced Ca^2+^ mobilisation in HEK-293 cells. Experimental conditions, in which the parameters were obtained, are given on the left side. Cell and ER area, areas of the respective cross-sections in LSM images; ER area/cell area, ratio of ER to cell areas; TG, thapsigargin; IONO, ionomycin; b[Ca^2+^]_cyt_, basal cytosolic Ca^2+^; b[Ca^2+^]_ER_, basal ER Ca^2+^; pΔ[Ca^2+^]_cyt_, peak amplitude of Ca^2+^ transients; AUC, area under the curve; k_leak_, ER Ca^2+^ leak rate; τ_leak_, time constant of ER Ca^2+^ leak; k_depl_, ER Ca^2+^ depletion rate; τ_depl_, time constant of ER Ca^2+^ depletion; k_clear_, Ca^2+^ clearance rate; τ_clear_, time constant of cytosolic Ca^2+^ clearance. Data on the right side is presented as mean ± SEM. Number of cells: 21–472.

Cell and ER dimensions				
			Cell area	618.43 ± 26.34 µm^2^
			ER area	376.05 ± 14.84 µm^2^
			ER area/cell area	61.62 ± 0.82 %
Basal Ca^2+^ levels				
zero [Ca^2+^]_ext_			b[Ca^2+^]_cyt_	62.90 ± 0.79 nM
			b[Ca^2+^]_ER_	370.59 ± 35.60 µM
1 mM [Ca^2+^]_ext_			b[Ca^2+^]_cyt_	67.76 ± 0.94 nM
10 mM [Ca^2+^]_ext_			b[Ca^2+^]_cyt_	97.04 ± 2.67 nM
Cytosolic Ca^2+^ transients				
zero [Ca^2+^]_ext_/1 μM TG			pΔ[Ca^2+^]_cyt_	0.56 ± 0.01 µM
			AUC	133.31 ± 1.14 μM⋅s
1 mM [Ca^2+^]_ext_/1 μM TG			pΔ[Ca^2+^]_cyt_	1.06 ± 0.01 µM
			AUC	474.21 ± 4.32 μM⋅s
10 mM [Ca^2+^]_ext_/1 μM TG			pΔ[Ca^2+^]_cyt_	1.61 ± 0.05 µM
			AUC	675.73 ± 14.41 μM⋅s
Total cellular Ca^2+^ content				
zero [Ca^2+^]_ext_/10 µM IONO			pΔ[Ca^2+^]_cyt_	2.11 ± 0.04 µM
			AUC	108.15 ± 1.59 μM⋅s
ER and non-ER Ca^2+^ content				
zero [Ca^2+^]_ext_/1 μM TG			pΔ[Ca^2+^]_cyt_	0.52 ± 0.01 µM
			AUC	116.02 ± 1.43 μM⋅s
zero [Ca^2+^]_ext_/10 µM IONO			pΔ[Ca^2+^]_cyt_	0.23 ± 0.01 µM
			AUC	50.25 ± 0.51 μM⋅s
ER Ca^2+^ depletion				
zero [Ca^2+^]_ext_/1 μM TG			k_depl_	6.12 e−3 ± 0.97 e−3 s^−1^
			τ_depl_	162.41 ± 7.81 s
ER Ca^2+^ efflux (Ca^2+^ leak)				
zero [Ca^2+^]_ext_/1 μM TG			k_leak_	15.41 e−3 ± 2.53 e−3 s^−1^
			τ_leak_	77.48 ± 8.06 s
External Ca^2+^ influx (SOCE)				
1 mM [Ca^2+^]_ext_/1 μM TG			pΔ[Ca^2+^]_cyt_	1.85 ± 0.04 µM
10 mM [Ca^2+^]_ext_/1 μM TG			pΔ[Ca^2+^]_cyt_	3.41 ± 0.05 µM
Cytosolic Ca^2+^ clearance				
zero [Ca^2+^]_ext_/2 mM EGTA			k_clear_	28.34 e−3 ± 1.02 e−3 s^−1^
			τ_clear_	37.08 ± 1.43 s

Quantification of TG-induced Ca^2+^ transients in HEK-293 cells reveals a remarkable proportionality between the amplitude of the Ca^2+^ transients (Δ[Ca^2+^]_cyt_) and [Ca^2+^]_ext_ (Table 1). For instance, the peak amplitude of the Ca^2+^ transients (pΔ[Ca^2+^]_cyt_) increased by approx. 0.5 µM by increasing external Ca^2+^ from zero to 1 mM and, again by approx. 0.5 µM from 1 to 10 mM external Ca^2+^. The area under the curve (AUC) also followed the increases of [Ca^2+^]_ext_ (Table 1). However, a precise measure of the AUC is hampered by the long Ca^2+^ plateaus observed with 1 and 10 mM external Ca^2+^ (Figure 2A). Hence, our results suggest that the entry of external Ca^2+^ adds to the Ca^2+^ release from intracellular stores to build up the TG-induced Ca^2+^ mobilisation in HEK-293 cells (Figure 2A), similarly as described previously for other cell types (Fierro et al., 1998; Chen et al., 2003; Baggaley et al., 2008). Additionally, we regularly observed that the TG-induced transients in HEK-293 cells decayed to basal levels within 350–550 s in the absence of external Ca^2+^ (Figure 2A), indicating that the plasma membrane of HEK-293 cells has definitely the capacity to remove cytosolic Ca^2+^, as described for other cell types (see Putney, 2017). The observations made in the experiments shown in Figure 2A allowed us, therefore, to formulate the balance equation describing the changes in cytosolic Ca^2+^ (d[Ca^2+^]_cyt_/dt) induced by TG in HEK-293 cells:

$${d\left[{Ca}^{2+}\right]}_{cyt}/dt={J_{entry}+J}_{leak}-J_{clear}$$ (1)

where J_entry_, J_leak_ and J_clear_ denote the Ca^2+^ influx from the external space (SOCE), the Ca^2+^ efflux from internal stores (ER Ca^2+^ leak) and the Ca^2+^ efflux from cytosol due to clearance mechanisms, respectively. A similar approach has been used to model the Ca^2+^ dynamics in various cell types (Bergling et al., 1998; Perez-Rosas et al., 2015; Han et al., 2017).

### 3.3 The Ca^2+^ content of HEK-293 cells

Ca^2+^ is stored in the ER, mitochondria, and Golgi apparatus, whereby the ER is believed to be the main Ca^2+^ storage organelle in mammalian cells (Wang et al., 2019). Using [Ca^2+^]_cyt_ imaging, we next estimated the total amount of releasable Ca^2+^ that is stored in HEK-293 cells (Figure 2B). For this purpose, we took advantage of ionomycin (IONO), a Ca^2+^ ionophore that releases Ca^2+^ unselectively from ER, mitochondria, and other cell compartments (Mckenzie and Duchen, 2016; Pick et al., 2021). These experiments were performed in the absence of external Ca^2+^ to prevent contamination by the entry of external Ca^2+^. As shown in Figures 2A, B and Table 1, IONO mobilised in general more Ca^2+^ than TG in the absence of external Ca^2+^. In average the IONO-induced Ca^2+^ transients were approx. 4 times higher than those observed after TG-application in Ca^2+^-free recording solution. The IONO-induced Ca^2+^ transients were also short and, therefore, the AUC values were slightly lower than those of TG-induced transients. In order to precise the contribution of ER and non-ER Ca^2+^ stores to the total content of releasable Ca^2+^ in HEK-293 cells, we modified our protocol and applied TG followed by IONO (Figure 2C). In this protocol, the first Ca^2+^ surge induced by TG largely reflects the release of Ca^2+^ from the ER, i.e., from ER Ca^2+^ stores. The second Ca^2+^ surge induced by IONO arises from the release of Ca^2+^ sequestered in the remaining stores, which we termed non-ER Ca^2+^ stores. It is believed that Ca^2+^ released from mitochondria dominates during this IONO-induced Ca^2+^ surge (Mckenzie and Duchen, 2016; Pick et al., 2021). Comparing the amplitude and AUC of the TG- and IONO-induced Ca^2+^ transient shown in Figure 2C, it appears that the amount of Ca^2+^ released from ER is twice the amount of releasable Ca^2+^ present in non-ER stores of HEK-293 cells (Table 1).

### 3.4 The Ca^2+^ entry and clearance in HEK-293 cells

The comparison of the Ca^2+^ mobilisation induced by TG in the presence and absence of external Ca^2+^ revealed SOCE in HEK-293 cells (Figure 2A). In order to measure SOCE separately, we used the standard Ca^2+^ re-addition protocol, which consisted in emptying Ca^2+^ stores with TG in nominally Ca^2+^-free solutions and raise external Ca^2+^ immediately after full Ca^2+^ depletion (see Prakriya and Lewis, 2015). In our experiments, we assessed SOCE as the amplitude of the cytosolic Ca^2+^ surges observed after re-addition of 1 and 10 mM external Ca^2+^ (Figure 2D). Such SOCE-induced transients were proportional to [Ca^2+^]_ext_ (Table 1). Compared with the Ca^2+^ transients shown in Figure 2A, the SOCE-induced Ca^2+^ transients were much higher. Using the Ca^2+^ re-addition data, we calculated that SOCE produced an increase of 29.25 ± 0.86 nM⋅s^−1^ and 51.42 ± 1.29 nM⋅s^−1^ in [Ca^2+^]_cyt_ when HEK-293 cells were abruptly exposed to 1 and 10 mM external Ca^2+^, respectively. Similar measurements with 0.5–2.0 mM external Ca^2+^ in other cell lines have revealed much smaller SOCE-induced Ca^2+^ transients (Feske et al., 2005; Li et al., 2018; Merino-Wong et al., 2021; Pick et al., 2021; Martínez-Martínez et al., 2022). Thus, it appears that our HEK-293 cells express a prominent SOCE. However, such high SOCE expression may be clone-specific because a high clone-to-clone variability of SOCE expression has been reported for the HEK-293 cell line (Zagranichnaya et al., 2005).

As illustrated by the cytosolic Ca^2+^ transients shown in Figures 2A–D, Ca^2+^ clearance mechanisms must exist to limit surges in [Ca^2+^]_cyt_ and, therefore, almost all Ca^2+^ transients in HEK-293 cells tended to decay quickly toward basal [Ca^2+^]_cyt_ levels. To measure the capacity of the plasma membrane to remove cytosolic Ca^2+^, we used the standard Ca^2+^ clearance assay (see Baggaley et al., 2008). As shown in Figure 2E, we first added Ca^2+^ to the external solution and initiated SOCE to raise [Ca^2+^]_cyt_ in cells that were previously treated with TG. At the peak of the SOCE-induced Ca^2+^ surge, we next chelated external Ca^2+^ with EGTA and interrupted the Ca^2+^ entry. As a consequence, [Ca^2+^]_cyt_ decayed rapidly reflecting the clearance of cytosolic Ca^2+^ (Figure 2E). As shown in Figure 2F, the decay of [Ca^2+^]_cyt_ after chelation of external Ca^2+^ is linear in the semi-log plot, suggesting that it follows an exponential time course. Accordingly, we assume that the Ca^2+^ clearance follows a first order kinetics in HEK-293 cells, and it can be described by the equation

$${d\left[{Ca}^{2+}\right]}_{cyt}/dt=-k_{clear}∙{\left[{Ca}^{2+}\right]}_{cyt}\;\left(t\right)$$ (2)

where k_clear_ represents the Ca^2+^ clearance rate. By integration of Eq. 2, we obtained the equation that describes the time course of [Ca^2+^]_cyt_ after unmasking the Ca^2+^ clearance.

$${\left[{Ca}^{2+}\right]}_{cyt}\left(t\right)={\left[{Ca}^{2+}\right]}_{cyt}\left(0\right)∙\exp\left(-k_{clear}∙t\right)$$ (3)

[Ca^2+^]_cyt_ (0) corresponds to the Ca^2+^ concentration at the time point when SOCE was abruptly interrupted. In order to facilitate the interpretation of our data, we also calculated τ_clear_, the time constant for Ca^2+^ clearance that is defined as the inverse of k_clear_. Figure 2F illustrates the fitting of Eq. 3 to the data. The average values of k_clear_ and τ_clear_ are presented in Table 1. Using Eq. 2 and the mean value of k_clear_ as given in Table 1, it can be calculated that the decay in [Ca^2+^]_cyt_ due to the Ca^2+^ clearance is around 10–13 nM⋅s^−1^ for mid values of [Ca^2+^]_cyt_ (0.35–0.45 µM). This Ca^2+^ clearance rate is much lower than described for other cell types (Chen et al., 2003; Baggaley et al., 2008; Pick et al., 2021) and may be characteristic for HEK-293 cells. Since we obtained our data in the presence of TG, we have no information on the Ca^2+^ clearance mediated by SERCA pumps. The parameters given in Table 1 most likely reflect the Ca^2+^ clearance *via* the plasma membrane of HEK-293 cells.

### 3.5 Parallel changes of cytosolic and ER Ca^2+^ in HEK-293 cells

**FIGURE 3:**
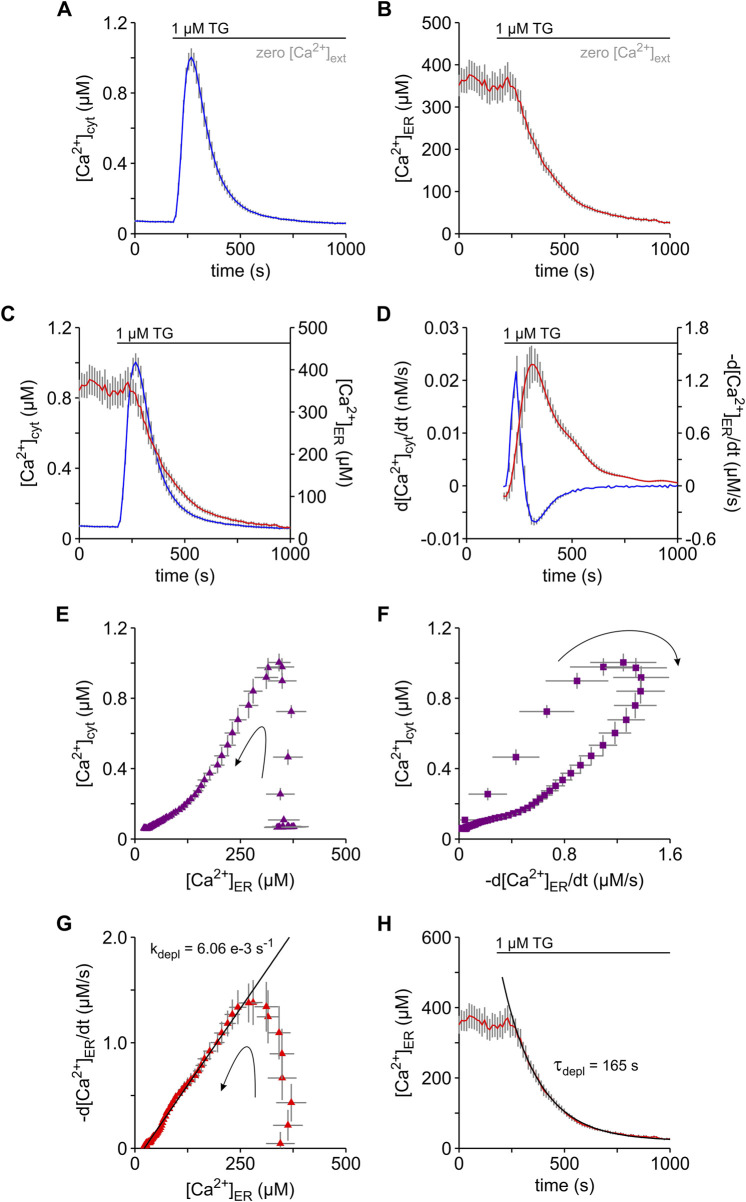
Analysis of the Ca^2+^ depletion in the ER and its impact on cytosolic Ca^2+^ levels of HEK-D1ER cells. Simultaneous imaging of cytosolic Ca^2+^ ([Ca^2+^]_cyt_) and ER Ca^2+^ ([Ca^2+^]_ER_) were performed with FURA-2 and D1ER in the same cells, respectively. For this purpose, we used the HEK-D1ER cell line that stably expresses the genetically encoded ER Ca^2+^ sensor D1ER. To prevent entry of external Ca^2+^, the experiments were carried out in the absence of external Ca^2+^ (zero [Ca^2+^]_ext_). **(A–C)** Thapsigargin (1 μM TG) induced a surge in [Ca^2+^]_cyt_ **(A)** that timely correlated with a decrease in [Ca^2+^]_ER_ **(B)**. [Ca^2+^]_cyt_ and [Ca^2+^]_ER_ are superimposed to illustrate the correlation **(C)**. **(D)** Temporal changes of cytosolic and ER Ca^2+^ are shown as the first derivatives of [Ca^2+^]_cyt_ (d[Ca^2+^]_cyt_/dt, blue) and [Ca^2+^]_ER_ (-d[Ca^2+^]_ER_/dt, red), respectively. **(E,F)** The dependence of [Ca^2+^]_cyt_ on [Ca^2+^]_ER_ **(E)** and -d[Ca^2+^]_ER_/dt **(F)** strengthens the impact of the Ca^2+^ leak from ER on cytosolic Ca^2+^. **(G,H)** The decaying phase in the plot of d[Ca^2+^]_ER_/dt vs. [Ca^2+^]_ER_ follows a linear relation as expected for a first order kinetic with a constant leak rate (k_depl_) of 6.06 e−3 s^−1^ **(G)**. Accordingly, an exponential function with a time constant (τ_depl_) of 165 s, which reflects the invers of k_depl_, best fits the time course of the TG-induced decay of [Ca^2+^]_ER_ **(F)**. Protocols of TG application are depicted above the graphs. Arrows indicate the time course of events **(E–G)**. Data is given as mean ± SEM. Number of cells: 35.

As in almost every cell type that has been tested (Thastrup et al., 1990; Treiman et al., 1998), TG-induced cytosolic Ca^2+^ transients in HEK-293 cells (Figure 2A). Specifically, the release of Ca^2+^ from the ER was sufficient to generate cytosolic Ca^2+^ transients because such transients were readily recorded in the absence of external Ca^2+^. In order to understand the mechanism behind this phenomenon, we recorded [Ca^2+^]_cyt_ and [Ca^2+^]_ER_ simultaneously using FURA-2 and the genetically encoded ER Ca^2+^ sensor D1ER, respectively (Figure 3). For this purpose, we used the HEK-D1ER cell line that was generated by stable transfection of HEK-293 cells with D1ER (Gamayun et al., 2019). All experiments shown in Figure 3 were performed in the absence of external Ca^2+^. Under these conditions, basal ER Ca^2+^ levels of HEK-D1ER cells (b[Ca^2+^]_ER_) scattered between 300 and 500 µM with a mean of approx. 370 μM (Table 1), which is slightly lower than previous measurements of basal ER Ca^2+^ in various cell lines (Suzuki et al., 2014). The b[Ca^2+^]_cyt_ of HEK-D1ER cells was within the range observed in HEK-293 cells. As expected, TG induced the typical surge in [Ca^2+^]_cyt_ in HEK-D1ER cells (Figure 3A). Although the simultaneous imaging of ER and cytosolic Ca^2+^ was performed in the absence of external Ca^2+^, the pΔ[Ca^2+^]_cyt_ was 0.91 ± 0.05 µM in the experiments with HEK-D1ER cells shown in Figure 3A. Generally, the pΔ[Ca^2+^]_cyt_ of HEK-D1ER cells scattered between 0.7 and 1.0 µM and, therefore, it was much higher than in the native HEK-293 cells (Table 1). We presume that this difference is associated with the inevitable cell cloning that we performed when the HEK-D1ER cell line was established.

In parallel to the cytosolic Ca^2+^ surge, we observed a decay in [Ca^2+^]_ER_ after TG application (Figure 3B), as previously reported for various cell types (Palmer et al., 2004; Zima et al., 2010; Suzuki et al., 2014; Bovo et al., 2017; Leon-Aparicio et al., 2017; Gamayun et al., 2019; Bhadra et al., 2021). When the time courses of [Ca^2+^]_cyt_ and [Ca^2+^]_ER_ were compared, it became clear that the cytosolic Ca^2+^ surge reached a peak before substantial ER Ca^2+^ depletion was detectable (Figure 3C). This apparent absence of correlation between [Ca^2+^]_cyt_ and [Ca^2+^]_ER_ has been previously reported (Leon-Aparicio et al., 2017; Gamayun et al., 2019). To understand the impact of ER Ca^2+^ release on [Ca^2+^]_cyt_, we next compared the first time-derivatives of [Ca^2+^]_cyt_ and [Ca^2+^]_ER_, whereby the first time-derivative of [Ca^2+^]_ER_ was expressed in terms of -d[Ca^2+^]_ER_/dt for simplicity. As shown in Figure 3D, the first observation we made was that d[Ca^2+^]_cyt_/dt increased and decreased rapidly while -d[Ca^2+^]_ER_/dt still was in the rising phase. Approx. 90 s after application of TG, d[Ca^2+^]_cyt_/dt became zero. During this 90 s time period, [Ca^2+^]_ER_ declined from an average b[Ca^2+^]_ER_ of 370 µM to approx. 348 μM, corresponding to about 6% Ca^2+^ depletion in the ER. Accordingly, a steep increase of [Ca^2+^]_cyt_ was detected at [Ca^2+^]_ER_ above 348 µM in correlation plots of [Ca^2+^]_cyt_ vs. [Ca^2+^]_ER_ (Figure 3E). Furthermore, plots of [Ca^2+^]_cyt_ as a function of -d[Ca^2+^]_ER_/dt showed that the upstroke of the cytosolic Ca^2+^ surges took place before -d[Ca^2+^]_ER_/dt reached its maximal value (Figure 3F). Thus, our results suggest that a 6% ER Ca^2+^ depletion is sufficient to generate the upstroke of cytosolic Ca^2+^ surges that generally reached peak values of 0.6–1.0 µM in native HEK-293 as well as in HEK-D1ER cells. A second important observation in the analysis of first time-derivatives was that the maximum of -d[Ca^2+^]_ER_/dt almost coincided with the minimum of d[Ca^2+^]_cyt_/dt (Figure 3D). After this time point, d[Ca^2+^]_cyt_/dt followed a time course that mirrored that of -d[Ca^2+^]_ER_/dt. Thus, the time point at which -d[Ca^2+^]_ER_/dt reached the maximum represents an inflection point, which divides the TG-induced Ca^2+^ mobilisation in two phases. The cytosolic Ca^2+^ surge was built up in the early phase while the late phase was characterized by the decay of the Ca^2+^ surge. The corresponding average values of [Ca^2+^]_ER_ and [Ca^2+^]_cyt_ at this inflection point were 311 µM and 0.92 µM, respectively. When we analysed the relationship of -d[Ca^2+^]_ER_/dt to [Ca^2+^]_ER_ (Figure 3G), we observed that there was a sharp increase of -d[Ca^2+^]_ER_/dt at ER Ca^2+^ levels above 311 μM, corresponding to the early phase of the TG-induced Ca^2+^ mobilisation. We hypothesise that this early phase reflected the cumulating TG-inhibition of SERCA pumps that reached maximum at the maximum of -d[Ca^2+^]_ER_/dt. Hence, the decaying phase of -d[Ca^2+^]_ER_/dt shown in Figures 3D, G developed under a fully unmasked Ca^2+^ leak from ER. Since it has been suggested that changes in the SR Ca^2+^ concentration may not necessarily correspond to the amount of released Ca^2+^ (Perez-Rosas et al., 2015), however, we cautiously treated the process underlying the late phase of TG-induced Ca^2+^ mobilisation simply as ER Ca^2+^ depletion. In this late phase, there was a linear relationship between -d[Ca^2+^]_ER_/dt and [Ca^2+^]_ER_ (Figure 3G), suggesting that the ER Ca^2+^ depletion followed a first order kinetic, which is expressed as

$${d\left[{Ca}^{2+}\right]}_{ER}/dt=-k_{depl}∙{\left[{Ca}^{2+}\right]}_{ER}\;\left(t\right)$$ (4)

where k_depl_ represents the Ca^2+^ depletion rate. A fit of Eq. 4 to the data revealed k_depl_ values around a mean of 6.12 e−3 s^−1^ for HEK-D1ER cells (Figure 3G; Table 1). By integration of Eq. 4, we generated the equation describing the time course of [Ca^2+^]_ER_ in the late phase of the TG-induced Ca^2+^ mobilisation.

$${\left[{Ca}^{2+}\right]}_{ER}\left(t\right)=b{\left[{Ca}^{2+}\right]}_{ER}∙\exp\left(-k_{depl}∙t\right)$$ (5)

where b[Ca^2+^]_ER_ represents the basal [Ca^2+^]_ER_. As shown in Figure 2H, Eq. 5 nicely fits the time course [Ca^2+^]_ER_ in the late phase, i.e., after the inflection point of Ca^2+^ mobilisation. To allow comparison to published data, we calculated the time constant of Ca^2+^ depletion (τ_depl_) that is defined as the inverse of k_depl_ (Table 1). Although τ_depl_ is frequently used in models of Ca^2+^ dynamics as a correlate of the Ca^2+^ leak from ER (Bergling et al., 1998; Means et al., 2006), few reports provide quantitative data. Our τ_depl_ estimate of approx. 165 s for HEK-D1ER cells fits within the published data on ER Ca^2+^ depletion in various cell lines (Lomax et al., 2002; Gamayun et al., 2019; Bhadra et al., 2021).

### 3.6 Pharmacological modulation of the Ca^2+^ mobilisation in HEK-293 cells

**FIGURE 4:**
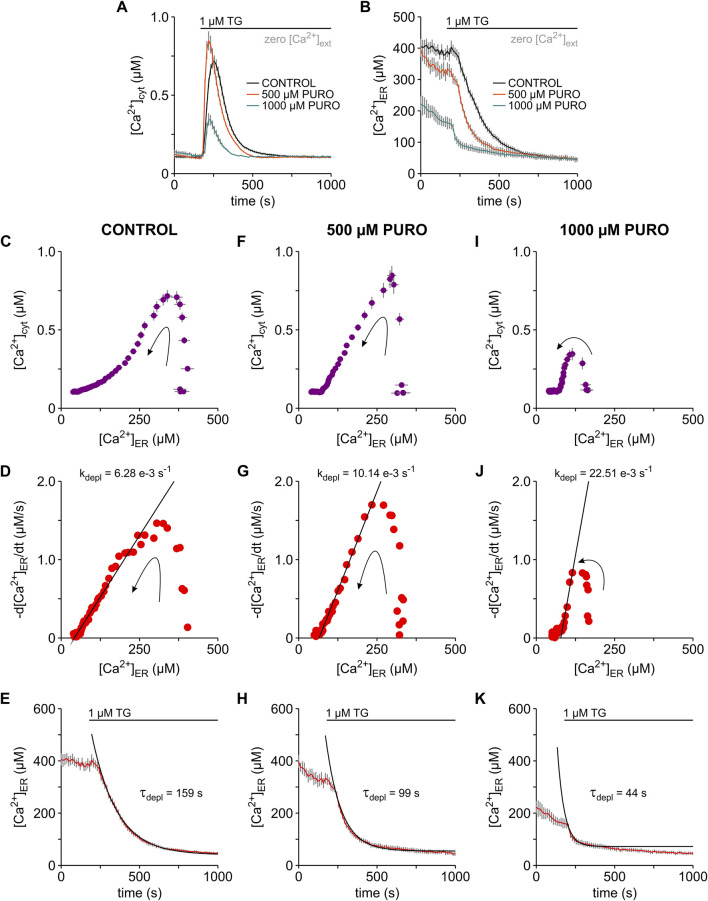
Puromycin effects on the TG-induced Ca^2+^ mobilisation in HEK-D1ER cells. Cytosolic Ca^2+^ ([Ca^2+^]_cyt_) and ER Ca^2+^ ([Ca^2+^]_ER_) were imaged simultaneously with FURA-2 and D1ER in the HEK-D1ER cell line. Cells were exposed to 500 and 1000 µM puromycin (500 µM PURO, 1000 µM PURO) for 10 min in a solution containing 2 mM Ca^2+^ (not shown). Control treatment (CONTROL) was carried out just with the 2 mM Ca^2+^ solution. Ca^2+^ imaging recordings were performed in the absence of external Ca^2+^ (zero [Ca^2+^]_ext_). Time courses of [Ca^2+^]_cyt_ **(A)** and [Ca^2+^]_ER_ **(B)** in control cells and after treatment with 500 μM and 1000 µM PURO. For control, 500 and 1000 µM PURO treatments, graphs show the correlation between [Ca^2+^]_cyt_ and [Ca^2+^]_ER_ **(C,F,I)**, the dependence of the first derivative of the ER Ca^2+^ concentration (-d[Ca^2+^]_ER_/dt) on [Ca^2+^]_ER_ **(D,G,J)** and the time course of the TG-induced ER Ca^2+^ depletion **(E,H,K)**. The decaying phases in the plots shown in **(D,G,J)** were fitted with linear relations (black lines), in which the leak rates (k_depl_) were 6.28 e−3, 10.14 e−3 and 22.51 e−3 s^−1^, respectively. τ_depl_ values of 159, 99 and 44 s were calculated as the inverse of the corresponding k_depl_. Exponential functions (black lines) were constructed with the calculated τ_depl_ values and superimposed on the time courses of ER Ca^2+^ depletion, as shown in **(E,H,K)**. Protocols of TG application are depicted above the graphs. Arrows indicate the time course of events **(C,D,F,G,I,J)**. Data is given as mean ± SEM. Number of cells: 12–16 cells.

Several studies have analysed the action of pharmacological active substances on the ER Ca^2+^ leak mediated by Sec61 translocons mostly by measuring the effects of these substances on TG-induced cytosolic Ca^2+^ transients (see Parys and van Coppenolle, 2022). Seminal work with tunicamycin, dithiothreitol (DTT), trifluoperazine and ophiobolin A has shown that these substances enhance TG-induced cytosolic Ca^2+^ transients by increasing specifically the Ca^2+^ leak through Sec61 translocons (Erdmann et al., 2011; Schäuble et al., 2012). In line with this body of evidence, emetine, cycloheximide and anisomycin, which stabilize Sec61 translocons in a closed state (e.g., Al-Mawla et al., 2020), reduced the amplitude of TG-induced cytosolic Ca^2+^ transients (van Coppenolle et al., 2004; Hammadi et al., 2013; Klein et al., 2018; Pick et al., 2021). However, PURO, which favours the open, Ca^2+^-permeable state of Sec61 translocons, surprisingly reduced the amplitude of TG-induced cytosolic Ca^2+^ transients (van Coppenolle et al., 2004; Lang et al., 2011b). Likely, the increase in ER Ca^2+^ leak leads inevitably to ER Ca^2+^ depletion, which in turn attenuates cytosolic Ca^2+^ transients. This concept has been tested with eeyarestatins and trifluoperazine, which favour the open, Ca^2+^-permeable state of Sec61 translocons (Gamayun et al., 2019; Bhadra et al., 2021). In the case of eeyarestatins, an increase of the concentration switched the effects on TG-induced cytosolic Ca^2+^ transients from amplification to attenuation (Gamayun et al., 2019). A similar switch was produced by prolonging the exposure time to trifluoperazine (Bhadra et al., 2021). Based on these observations, we hypothesised that the amplification-to-attenuation switch induced by Sec61 modulators on TG-induced cytosolic Ca^2+^ transients is both time and concentration dependent. In order to test this hypothesis for PURO, we next exposed HEK-D1ER cells to 500 μM and 1000 µM PURO and analysed the effects on Ca^2+^ mobilisation using our simultaneously image system for [Ca^2+^]_cyt_ and [Ca^2+^]_ER_ (Figure 4). Unlike previous reports (e.g., Lang et al., 2011b), HEK-D1ER cells were exposed to PURO for 10 min in a bath solution containing 2 mM Ca^2+^ before Ca^2+^ imaging in zero external Ca^2+^ solutions. In our hands, the presence of Ca^2+^ in the external solution protects against a fast ER Ca^2+^ loss. In these experiments, the most obvious effect was an increase in the amplitude of the cytosolic TG-induced Ca^2+^ transients to 110–135% in cells treated with 500 µM PURO, while Ca^2+^ transients were dramatically reduced to 15–38% after exposure to 1000 µM PURO, when compared to controls (Figure 4A). Additionally, it is remarkable that the Ca^2+^ transients in cells exposed to PURO were shorter than in control cells. PURO also decreased ER Ca^2+^ levels and accelerated the ER Ca^2+^ depletion in HEK-D1ER cells (Figure 4B). In cells exposed to 500 µM PURO, there was a mild decrease of b[Ca^2+^]_ER_ to 80–91% compared to controls probably because PURO exposures were performed in the presence of external Ca^2+^. However, b[Ca^2+^]_ER_ decreased dramatically to 37–45% in cells exposed to 1000 µM PURO. Thus, the simultaneous imaging of [Ca^2+^]_cyt_ and [Ca^2+^]_ER_ in HEK-D1ER cells showed that PURO enhances TG-induced cytosolic Ca^2+^ transients as long as ER Ca^2+^ levels are not strongly compromised. When ER Ca^2+^ levels are low, the cytosolic Ca^2+^ transients became understandably smaller. On this basis, we assume that previously reported low amplitudes of the cytosolic Ca^2+^ transients reflect a considerable loss of ER Ca^2+^ induced by PURO most likely due to the absence of external Ca^2+^ during the PURO exposure (e.g., Lang et al., 2011b; Al-Mawla et al., 2020).

As shown in the plot of [Ca^2+^]_cyt_ vs. [Ca^2+^]_ER_ (Figures 4C, F), the increase of cytosolic Ca^2+^ at basal ER Ca^2+^ levels was particularly steep, indicating that the Ca^2+^ efflux from ER was enhanced by the treatment with 500 µM PURO. Large Ca^2+^ effluxes were not expected after treatment with 1000 µM PURO because basal ER Ca^2+^ levels were reduced under these conditions. Hence, the changes induced by TG in [Ca^2+^]_cyt_ were relatively small at this high PURO concentration (Figure 4I). When we calculated k_depl_ in plots of -d[Ca^2+^]_ER_/dt vs. [Ca^2+^]_ER_ (Figures 4D, G, J), the linear phases were generally much steeper in PURO-treated HEK-D1ER cells. Accordingly, k_depl_ increased from 6.51 e−3 ± 0.43 e−3 s^−1^ in control cells to 9.89 e−3 ± 0.75 e−3 s^−1^ and 22.46 e−3 ± 1.25 e−3 s^−1^ in HEK-D1ER cells treated with 500 and 1000 µM PURO, respectively (*p* < 0.01; number of cells: 8–16). This changes in the k_leak_ translated into faster ER Ca^2+^ depletion in PURO-treated cells compared to controls (Figures 4E, H, K). The time constant τ_depl_ decreased from 157.21 ± 4.34 s in control cells to 92.62 ± 7.85 s and 41.36 ± 6.27 s in HEK-293 cells treated with 500 and 1000 µM PURO, respectively (*p* < 0.01; number of cells: 8–16). Thus, PURO likely enhanced the ER Ca^2+^ leak from ER, which produced a decrease of ER Ca^2+^ levels already during the exposure and then accelerated the Ca^2+^ depletion in ER when the Ca^2+^ leak was unmasked with TG. As a consequence, the effects on cytosolic TG-induced Ca^2+^ transients were sharply dependent on the PURO concentration. At the low PURO concentration of 500 μM, we observed an increase of the amplitude of the cytosolic Ca^2+^ transients, when the increase in k_depl_ was moderate. At the concentration of 1000 µM PURO, the loss of ER Ca^2+^ dominated and the cytosolic TG-induced Ca^2+^ transients became smaller than in control cells.

### 3.7 Modelling of Ca^2+^ transients induced by TG in HEK-293 cells

Most studies of the Ca^2+^ leak from ER have been performed in the absence of external Ca^2+^ to avoid SOCE (see Lomax et al., 2002; Lang et al., 2017; Parys and van Coppenolle, 2022). In this approach, the Ca^2+^ leak from ER is the only Ca^2+^ source underlying changes in [Ca^2+^]_cyt_. Hence, J_entry_ has not been included in further analysis and we simplified Eq. 1 to obtain the following balance equation that describes the time course of cytosolic Ca^2+^ transients under blockade of SERCA pumps with TG in the absence of external Ca^2+^:

$${d\left[{Ca}^{2+}\right]}_{cyt}/dt=J_{leak}-J_{clear}$$ (6)

**FIGURE 5:**
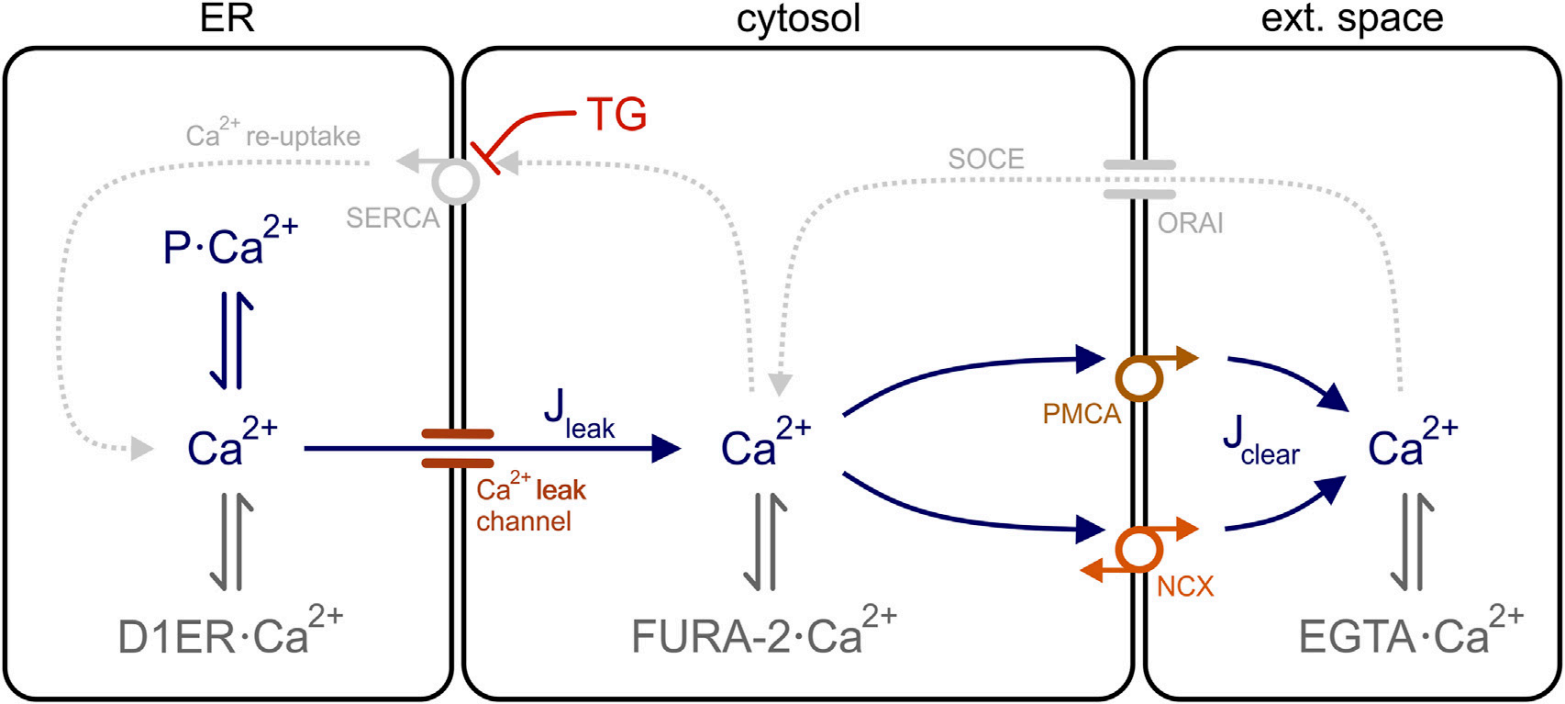
Model of the Ca^2+^ dynamics in HEK-293 cells. Three cell compartments are depicted in the model: ER, cytosol, and extracellular space. In the ER, free Ca^2+^ is in equilibrium with Ca^2+^ bound to the ER Ca^2+^ sensor D1ER (D1ER·Ca^2+^) and to luminal ER binding proteins (P·Ca^2+^), such as calreticulin and BiP. For simplicity, Ca^2+^ binds only to the Ca^2+^ sensor FURA-2 (FURA-2·Ca^2+^) and to the chelator EGTA (EGTA·Ca^2+^) in the cytosol an extracellular space, respectively. Ca^2+^ ions flow out of the ER through leak channels (J_leak_) and are removed from the cytosol *via* clearance mechanisms (J_clear_). In the presence of thapsigargin (TG), SERCA pumps are blocked and the Ca^2+^ re-uptake into the ER (Ca^2+^ re-uptake) is interrupted. The store-operated Ca^2+^ entry (SOCE) into the cells is also disrupted because Ca^2+^ is chelated in the external solution with EGTA. PMCA, plasma membrane Ca^2+^ ATPase; SERCA, sarco/endoplasmic reticulum Ca^2+^ ATPase, NCX, Na^+^-Ca^2+^ exchanger.

This equation implies that J_leak_ and J_clear_ are the only Ca^2+^ fluxes involved in the generation of cytosolic Ca^2+^ transients. Accordingly, the cytosol is the central compartment in our model of the Ca^2+^ dynamics of HEK-293 cells and is flanked by the ER and the extracellular space, whereby the ER functions as Ca^2+^ source and the extracellular space behaves as a Ca^2+^ sink because EGTA was present in the external solution (Figure 5). Therefore, no changes in [Ca^2+^]_ext_ are expected during Ca^2+^ mobilisation.

Since the upstroke of the TG-induced cytosolic Ca^2+^ transients occurred with a marginal decrease of ER Ca^2+^ (Figures 3C–E), we assume that k_depl_ does not reflect the ER Ca^2+^ leak that generates cytosolic Ca^2+^ transients. Instead, we describe the ER Ca^2+^ leak as follows:

$$J_{leak}=-{d\left[{Ca}^{2+}\right]}_{lER}/dt$$ (7)

where [Ca^2+^]_lER_ represents the ER Ca^2+^ concentration that drives the Ca^2+^ leak and may correspond to a luminal Ca^2+^ concentration close to the ER membrane. Assuming a first order kinetics, the changes in [Ca^2+^]_lER_ can be then describes as:

$${d\left[{Ca}^{2+}\right]}_{lER}/dt={-k}_{leak}∙{\left[{Ca}^{2+}\right]}_{lER}\;\left(t\right)$$ (8)

k_leak_ is the ER Ca^2+^ leak rate. By integrating Eq. 8, we obtained the following expression for the time course of [Ca^2+^]_lER_:

$${\left[{Ca}^{2+}\right]}_{lER}\left(t\right)=b{\left[{Ca}^{2+}\right]}_{lER}∙\exp\left(-k_{leak}∙t\right)$$ (9)

and combining Eqs 7–9, the expression describing the ER Ca^2+^ leak can be written as:

$$J_{leak}=b{\left[{Ca}^{2+}\right]}_{lER}∙k_{leak}∙\exp\left(-k_{leak}∙t\right)$$ (10)

Following a similar rationale, we defined the clearance of cytosolic Ca^2+^ as:

$$J_{clear}=-{d\left[{Ca}^{2+}\right]}_{cyt}/dt$$ (11)

and using Eq. 2, the expression describing the Ca^2+^ clearance flux can be written as:

$$J_{clear}=k_{clear}∙{\left[{Ca}^{2+}\right]}_{cyt}\;\left(t\right)$$ (12)

In order to derive an expression that describes TG-induced cytosolic Ca^2+^ transients, we first substituted J_leak_ (Eq. 10) and J_clear_ (Eq. 12) in Eq. 6 and the balance equation became:

$$d{\left[{Ca}^{2+}\right]}_{cyt}/dt=b{\left[{Ca}^{2+}\right]}_{lER}∙k_{leak}∙\exp\left(-k_{leak}∙t\right)-k_{clear}∙{\left[{Ca}^{2+}\right]}_{cyt}\;\left(t\right)$$ (13)

Next, the following expression that models cytosolic Ca^2+^ transients was generated by integration of Eq. 13:

$${\left[{Ca}^{2+}\right]}_{cyt}\left(t\right)={b\left[{Ca}^{2+}\right]}_{lER}∙\left[k_{leak}/\left(k_{clear}-k_{leak}\right)\right]∙\left[\exp\left(-k_{leak}∙t\right)-\exp\left(-k_{clear}∙t\right)\right]$$ (14)

However, this equation predicts a zero value for b[Ca^2+^]_cyt_. Therefore, we expressed Eq. 14 in terms of Δ[Ca^2+^]_cyt_ (t) that we calculated by subtracting b[Ca^2+^]_cyt_ from [Ca^2+^]_cyt_ (t). Accordingly, the general equation that models TG-induced cytosolic Ca^2+^ transients in the absence of external Ca^2+^ is:

$${∆\left[{Ca}^{2+}\right]}_{cyt}\left(t\right)={b\left[{Ca}^{2+}\right]}_{lER}∙\left[k_{leak}/\left(k_{clear}-k_{leak}\right)\right]∙\left[\exp\left(-k_{leak}∙t\right)-\exp\left(-k_{clear}∙t\right)\right]$$ (15)

This expression is the so-called “Bateman equation” that has been extensively used to model drug concentrations in blood plasma (Garrett, 1994; Macheras and Chryssafidis, 2020).

**FIGURE 6:**
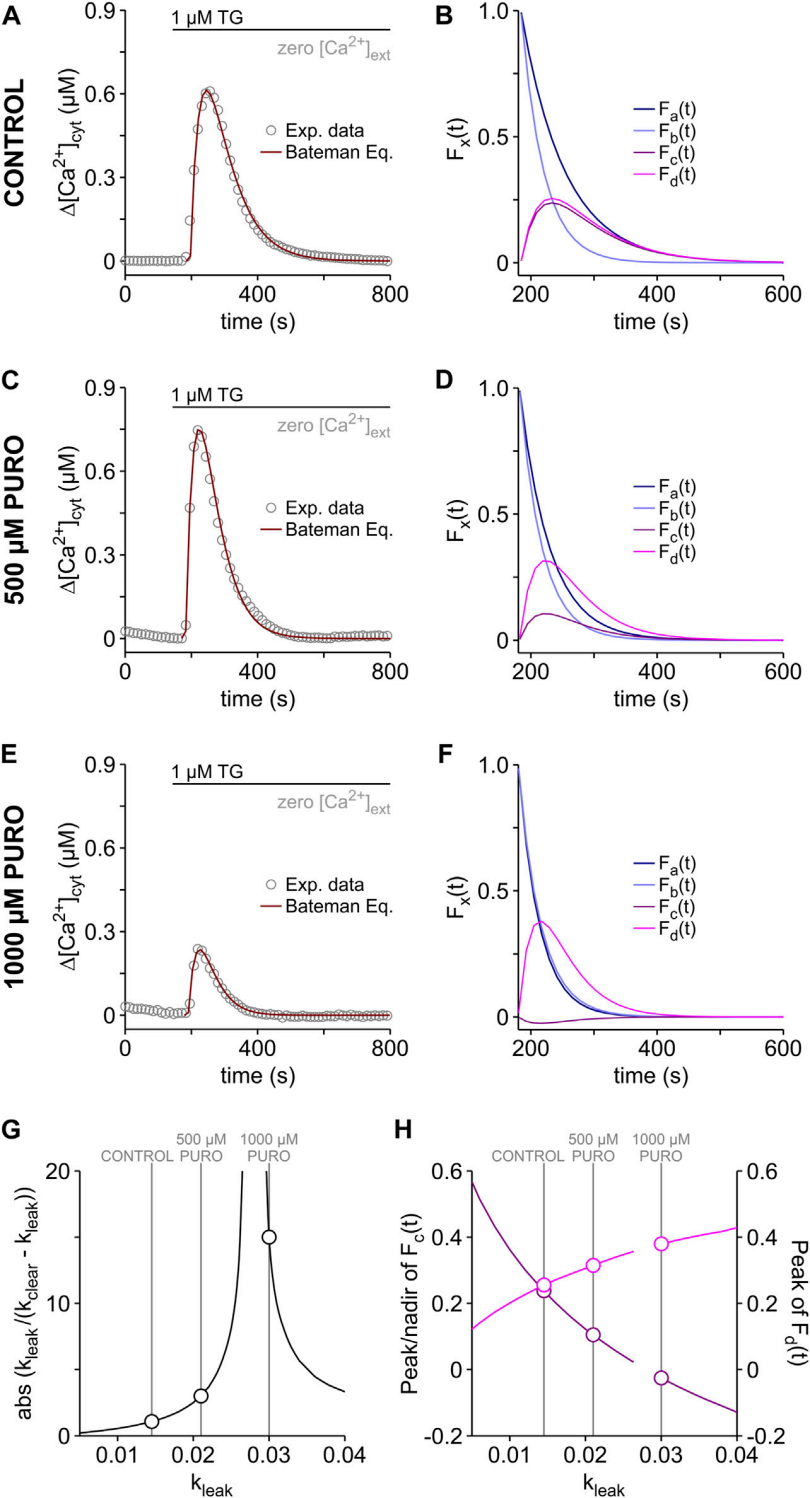
Reconstruction of TG-induced cytosolic Ca^2+^ transients. The Bateman equation (Eq. 15) was used to reconstruct the time course of the TG-induced Ca^2+^ transients recorded in HEK-D1ER cells treated with PURO and in the respective control (see Figure 4A). Shown are the reconstructed cytosolic Ca^2+^ transients superimposed on the experimental data **(A,C,E)**. PURO treatment protocol as in Figure 4A (CONTROL, 500 µM PURO and 1000 µM PURO). k_clear_ was 28.34 e−3 s^−1^ for all experimental conditions. k_leak_: **A**, 14.55 e−3 s^-1^; **C**, 21.03 e−3 s^−1^; **E**, 30.07 e−3 s^−1^. b[Ca^2+^]_lER_: **A**, 2.40 µM; **C**, 2.38 µM; **E**, 0.62 µM. In order to visualize the procedure of reconstruction, the Bateman equation was broken down in single terms as follows: F_a_(t) = exp (-k_leak_ ⋅ t); F_b_(t) = exp (-k_clear_ ⋅ t); F_c_(t) = exp (-k_leak_ ⋅ t) - exp (-k_clear_ ⋅ t) and F_d_(t) = (k_leak_/(k_clear_ - k_leak_)) ⋅ (exp (-k_leak_ ⋅ t) - exp (-k_clear_ ⋅ t)). The curves obtained in the breakdown of the Bateman equation are shown for controls **(B)** and for cells treated with 500 µM PURO **(D)** and 1000 µM PURO **(F)**. The dependence of the term [k_leak_/(k_clear_ - k_leak_)] on k_leak_ is shown in **(G)**. The peak and nadir values of F_c_(t) (dark pink) and F_d_(t) (pink) are depicted as a function of k_leak_ in **(H)**. Breaks in the curves of **(G,H)** are located around k_leak_ = k_clear_. The values for CONTROL, 500 µM PURO and 1000 µM PURO are highlighted in **(G,H)**.

The Bateman formalism assumes a one-compartment model, in which the ER Ca^2+^ leak and the clearance of cytosolic Ca^2+^ follow first-order kinetics (Eqs 2, 8). As a proof-of-principle, we applied the Bateman equation (Eq. 15) to reconstruct the TG-induced cytosolic Ca^2+^ transients measured in the PURO experiments (Figure 4). In these simulations, we assumed the same k_clear_ of 28.34 e−3 s^−1^ for all experimental conditions and the k_leak_ values were obtained by fitting Eq. 15 to the data (Figures 6A, C, E). As expected for an increase of ER Ca^2+^ leak, k_leak_ increased from 14.55 e−3 s^−1^ in control cells to 21.03 e−3 s^−1^ and 30.07 e−3 s^−1^ in cells exposed to 500 μM and 1000 µM PURO, respectively. Similar control k_leak_ values were obtained by fitting Eq. 15 to TG-induced Ca^2+^ transients of HEK-293 cells (Table 1). Interestingly, these k_leak_ values are approx. two times higher than those obtained for k_depl_ (Figures 4D, G, J). For comparison, we constructed Ca^2+^ transients with k_depl_ values and found that they were 50–100 s longer than the experimental counterparts (not shown). Thus, the cytosolic Ca^2+^ transients of HEK-D1ER cells reflected k_leak_ rather than k_depl_. On the other hand, the b[Ca^2+^]_lER_ values required to model the Ca^2+^ transients seem to mirror the PURO-induced changes in b[Ca^2+^]_ER_ (Figure 4B). The b[Ca^2+^]_lER_ values needed to fit cytosolic Ca^2+^ transients of control cells and of those treated with 500 µM PURO were very similar (2.38 µM vs. 2.40 µM, respectively), reflecting the minor loss of ER Ca^2+^ during the PURO treatment. The considerable loss of ER Ca^2+^ in cells treated with 1000 µM PURO (Figure 4B) was reflected in an b[Ca^2+^]_lER_ value that corresponded to approx. 25% of control (0.62 µM vs. 2.40 µM, respectively).

In general, it is expected that the shortest rate constant of the Bateman equation determines the decay of Ca^2+^ transients after its inflection point (see Garrett, 1994). Since k_leak_ < k_clear_ under control conditions (14.55 e−3 s^−1^ vs. 28.34 e−3 s^−1^), the term [exp (-k_leak_ ⋅ t) - exp (-k_clear_ ⋅ t)] of the Eq. 15 converges towards exp (-k_leak_ ⋅ t) in the tail of the Ca^2+^ transient, as shown in the breakdown of the Bateman equation (Figure 6B). A similar situation was found for the curves fitting the Ca^2+^ transient in the presence of 500 µM PURO (Figure 6D). In cells treated with 1000 µM PURO (Figure 6F), however, k_leak_ became slightly higher than k_clear_ (30.07 e−3 s^−1^ vs. 28.34 e−3 s^−1^). Therefore, it can be hardly distinguished whether the term [exp (-k_leak_ ⋅ t) - exp (-k_clear_ ⋅ t)] converge towards exp (-k_leak_ ⋅ t) or exp (-k_clear_ ⋅ t) in the tail of the Ca^2+^ transients (Figure 6F). Non-etheless, in general, the Bateman equation predicts that the decay of Ca^2+^ transient is dominated by k_clear_ if the relation of the constants reverses from k_leak_ < k_clear_ to k_leak_ > k_clear_ in the so-called flip-flop case (see Garrett, 1994). Thus, the decay of the TG-induced Ca^2+^ transients became faster in PURO-treated cells because k_leak_ increased compared to controls (Figures 6B, D, F) and consequently, the Ca^2+^ transients were shorter in PURO-treated cells (Figure 4A).

The peak amplitude of the Ca^2+^ transients increased by the treatment with 500 µM and decreased in the presence of 1000 µM PURO (Figure 4A), while the term [(k_leak_/(k_clear_ - k_leak_)) ⋅ (exp (-k_leak_ ⋅ t) - exp (-k_clear_ ⋅ t))] increased with the PURO treatment (Figures 6B, D, F). The latter is explained by the dependence of the term [k_leak_/(k_clear_ - k_leak_)] on k_leak_. Since [k_leak_/(k_clear_ - k_leak_)] is not determined when k_leak_ = k_clear_, the absolute value of [k_leak_/(k_clear_ - k_leak_)] increases asymptotically around k_leak_ = k_clear_, as shown in Figure 6G for k_clear_ = 28.34 e−3 s^−1^. Accordingly, the term [(k_leak_/(k_clear_ - k_leak_)) · (exp (-k_leak_ ⋅ t) - exp (-k_clear_ ⋅ t))] increases monotonically with k_leak_, although the term [exp (-k_leak_ ⋅ t) - exp (-k_clear_ ⋅ t)] decreases (Figure 6H). Hence, the amplitudes of the TG-induced Ca^2+^ transients increase theoretically when the ER Ca^2+^ leak is enhanced. Experimentally, however, this prediction holds as long as the ER Ca^2+^ content remains constant during the treatment with Ca^2+^ leak enhancers. Yet, the increasing Ca^2+^ leak begins to deplete the ER already during the period of incubation with enhancers of Ca^2+^ leak (Van Coppenolle et al., 2004; Gamayun et al., 2019; Al-Mawla et al., 2020). When TG is then applied, the depleted ER generates Ca^2+^ transients with smaller amplitudes than those arising from a full ER. As shown in Figures 4A, B, this was the case in cells treated with 1000 µM PURO and b[Ca^2+^]_lER_ rather than [k_leak_/(k_clear_ - k_leak_)] determined the amplitude of the cytosolic Ca^2+^ transients in these cells. Conversely, the mild loss of ER Ca^2+^ in cells treated with 500 µM PURO was apparently compensated by the increase in k_leak_ and the term [k_leak_/(k_clear_ - k_leak_)] dominated over b[Ca^2+^]_lER_ in setting the amplitude of the cytosolic Ca^2+^ transients. Thus, the magnitude of ER Ca^2+^ loss produced by Ca^2+^ leak enhancers dictates whether b[Ca^2+^]_lER_ or [k_leak_/(k_clear_ - k_leak_)] determines the amplitude of TG-induced cytosolic Ca^2+^ transients.

**FIGURE 7:**
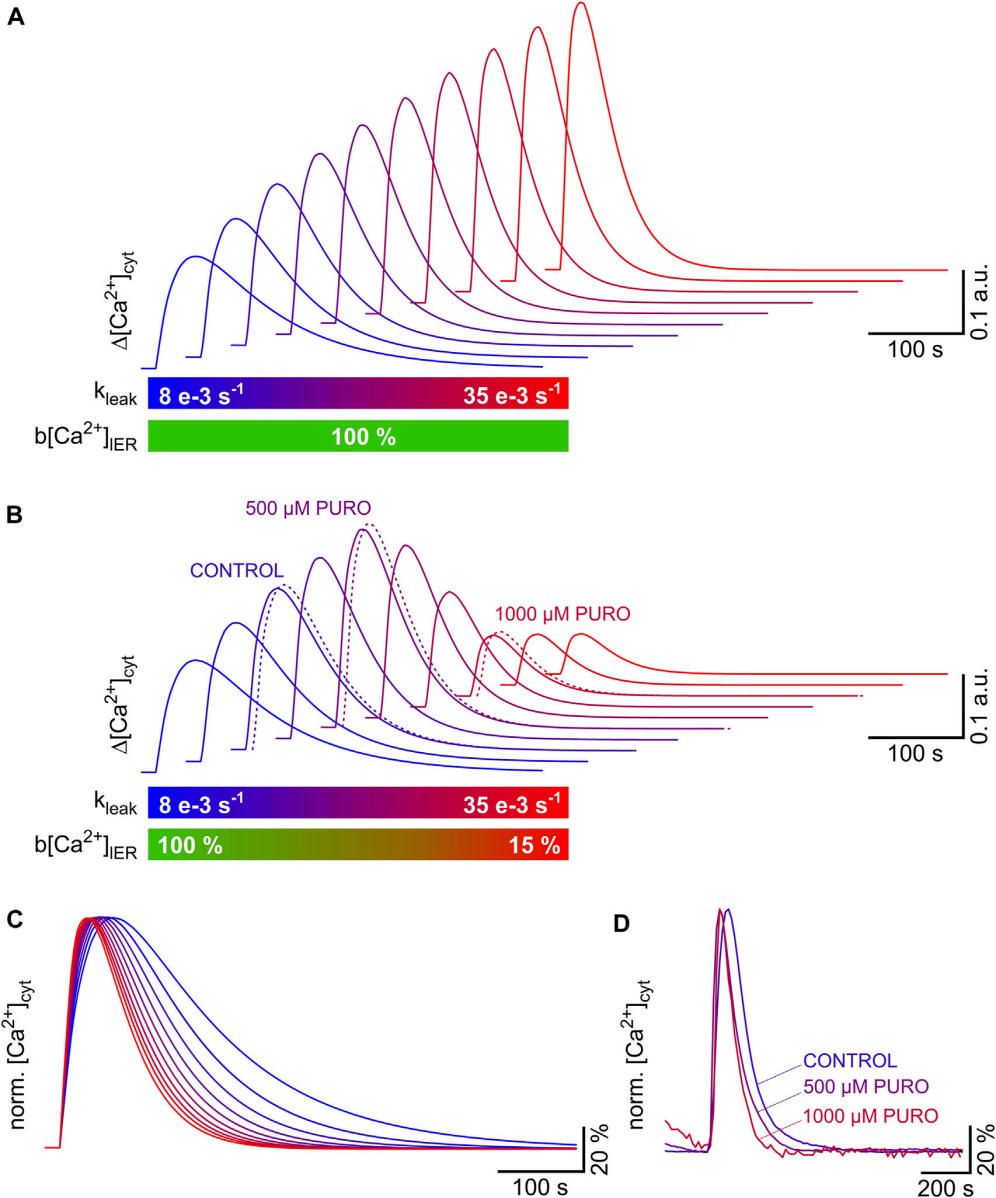
Amplification and attenuation of TG-induced cytosolic Ca^2+^ transients as a result of progressive increase in ER Ca^2+^ leak and loss of ER Ca^2+^ content. Cytosolic Ca^2+^ transients were modelled using the Bateman equation (Eq. 15). The increase in ER Ca^2+^ leak was simulated by raising k_leak_ from 8 e−3 s^−1^ to 35 e−3 s^−1^ in steps of 3 e−3 s^−1^. The Ca^2+^ clearance (k_clear_, 28 e−3 s^−1^) was assumed to be constant. ER Ca^2+^ levels (b[Ca^2+^]_lER_) were either kept constant or reduced from 100% to 15%. **(A,B)** A progressive amplification of the TG-induced cytosolic Ca^2+^ transients is evident when the ER Ca^2+^ leak increases and ER Ca^2+^ levels remain constant **(A)**. The inevitable loss of ER Ca^2+^, which follows the increase in Ca^2+^ leak, counteracts this amplification, and the process is eventually reversed resulting in the attenuation of the TG-induced cytosolic Ca^2+^ transients **(B)**. The switch from amplification to attenuation of TG-induced cytosolic Ca^2+^ transients was also observed in HEK-D1ER cells exposed to PURO (see Figure 4). To illustrate this phenomenon, cytosolic Ca^2+^ transients obtained in HEK-D1ER cells exposed to PURO are superimposed in **(B)** (dotted lines: CONTROL, 500 µM PURO, 1000 µM PURO). The parameters k_leak_ and b[Ca^2+^]_lER_ are colour coded below graphs in **(A,B)**. **(C,D)** The cytosolic Ca^2+^ transients shown in **(B)** were normalised to illustrate the fact that the duration and the time to peak of the TG-induced cytosolic Ca^2+^ transients are reduced as the ER Ca^2+^ leak increases **(C)** Same colour coding for k_leak_ as in **(B)**. The TG-induced cytosolic Ca^2+^ transients recorded in HEK-D1ER cells exposed to PURO were normalised (same experiments as in Figure 4A) to illustrate that the Ca^2+^ transients became faster and shorter with increasing PURO concentrations.

As shown in Figure 6, increases in the ER Ca^2+^ leak can amplify or attenuate the TG-induced cytosolic Ca^2+^ transients. The reason for this switch from amplification to attenuation of cytosolic Ca^2+^ transients is that the Ca^2+^ leak induces a loss of ER Ca^2+^, which in turns reduces the amplitude of the cytosolic Ca^2+^ transients. To illustrate the general principle of this phenomenon, we modelled TG-induced cytosolic Ca^2+^ transients using Eq. 15 with increasing k_leak_ values between 8 e−3 and 35 e−3 s^−1^ (Figures 7A–C). The clearance of cytosolic Ca^2+^ was assumed to be constant with a k_clear_ value of 28 e−3 s^−1^. ER Ca^2+^ levels were either maintained constant (Figure 7A) or an ER Ca^2+^ loss was modelled by reducing b[Ca^2+^]_lER_ values from 100% to 15% (Figure 7B). As it can be predicted from Figure 6H, the amplitude of the calculated cytosolic Ca^2+^ transients increased monotonically, i.e., there was a continuous amplification of the cytosolic Ca^2+^ transients when the ER Ca^2+^ levels were maintained constant (Figure 7A). However, it is more likely that the ER Ca^2+^ levels decrease as an inevitable consequence of the increasing Ca^2+^ leak, for instance, when the ER Ca^2+^ leak exceeds the pumping capacity of SERCA. The result of a decrease in ER Ca^2+^ levels on the top of an increasing ER Ca^2+^ leak is shown in Figure 7B. The calculated cytosolic Ca^2+^ transients were amplified by the initial increase of k_leak_ as long as ER Ca^2+^ levels were not compromised. Further increase of k_leak_ to high levels accompanied by strong ER Ca^2+^ loss attenuated the calculated cytosolic Ca^2+^ transients. This switch from amplification to attenuation correlated with changes in the shape of cytosolic Ca^2+^ transients. As illustrated by the normalised Ca^2+^ transients in Figure 7C, the calculated Ca^2+^ transients became shorter, and the rising phase became faster as k_leak_ progressively increased. ER Ca^2+^ levels had no effects on the shape of the Ca^2+^ transients that was determined only by k_leak_. Finally, we compared these calculated Ca^2+^ transients with the TG-induced cytosolic Ca^2+^ transients obtained in HEK-D1ER cells exposed to PURO (same experiments as in Figure 4). As illustrated in Figure 7B, the cytosolic Ca^2+^ transients of cells exposed to 500 µM PURO fit perfectly within the range of amplified Ca^2+^ transients, while those obtained after exposure to 1000 µM PURO correspond to attenuated Ca^2+^ transients. Compared to controls, the duration and rising phase of TG-induced cytosolic Ca^2+^ transients were shorter and faster, respectively, in HEK-D1ER cells exposed to PURO (Figure 7D). Thus, the experiments with HEK-D1ER cells exposed to PURO recapitulate the switch from amplification to attenuation of TG-induced cytosolic Ca^2+^ transients that results when the ER Ca^2+^ leak increases and ER Ca^2+^ levels decrease, as observed when Sec61 modulators enhance the ER Ca^2+^ leak through Sec61 translocons (Gamayun et al., 2019; Bhadra et al., 2021).

All in all, we described the cytosolic Ca^2+^ transients of HEK-293 cells using a simple one-compartment model, in which the ER Ca^2+^ leak and the cytosolic Ca^2+^ clearance followed first-order kinetics. Furthermore, we derived a Bateman equation that accounted for changes in the time course and amplitude of cytosolic Ca^2+^ transients that resulted when the ER Ca^2+^ leak was increased by PURO. In this model, the best indicators for an enhanced ER Ca^2+^ leak were the rise in amplitude accompanied by shortening of the duration of cytosolic Ca^2+^ transients, as long as the ER Ca^2+^ content was not compromised by the enhanced Ca^2+^ leak. In the case that the ER Ca^2+^ content was reduced by a strong Ca^2+^ leak, reduction of amplitude as well as shortening of cytosolic Ca^2+^ transients were the best indicators for an enhanced ER Ca^2+^ leak.

## 4 Discussion

In this work, we have studied the Ca^2+^ dynamics in the model cell line HEK-293. Firstly, we obtained morphological data on the ER of this cell line and then proceeded to image cytosolic and ER Ca^2+^ with FURA-2 and D1ER, respectively, to analyse quantitatively cell responses to TG and IONO. These experiments provided estimates of total Ca^2+^ content in the cells as well as on the Ca^2+^ content of ER and non-ER storage compartments. Using standard protocols, we further quantified SOCE and the Ca^2+^ clearance in HEK-293 cells. Next, we correlated the TG-induced Ca^2+^ depletion in ER with the respective cytosolic Ca^2+^ transients that we recorded in the absence of external Ca^2+^. On this basis, we were able to follow step-by-step how the cytosolic Ca^2+^ transients are built up after TG application. Finally, we reconstructed cytosolic Ca^2+^ transients using a one-compartment model, in which Ca^2+^ leak and Ca^2+^ clearance obey a first order kinetics. The Bateman equation, which is central to this model, reproduced the changes in amplitude and duration of TG-induced Ca^2+^ transients that we observed when the ER Ca^2+^ leak was increased by exposing HEK-D1ER cells to PURO.

Our quantitative analysis of the Ca^2+^ mobilisation in HEK-D1ER cells using simultaneous imaging of cytosolic and ER Ca^2+^ highlights two phases in the TG-induced Ca^2+^ mobilisation: an initial phase in which the surge in [Ca^2+^]_cyt_ is built up with a modest decrease in [Ca^2+^]_ER_ and a late phase in which both [Ca^2+^]_cyt_ and [Ca^2+^]_ER_ decrease in parallel (Figures 3C, D). The time point at which -d[Ca^2+^]_ER_/dt reached the peak represents the inflection point, which divides the TG-induced Ca^2+^ mobilisation in an early and a late phase. Besides ER Ca^2+^ leak and cytosolic Ca^2+^ clearance, other factors such as Ca^2+^ buffering in cytosol definitely also shape cytosolic Ca^2+^ transients (Means et al., 2006). Our simultaneous imaging of ER and cytosolic Ca^2+^ revealed another aspect specific for the TG action, i.e., the progressive inhibition of SERCA pumps in the ER. Since TG is applied extracellularly, it crosses the plasma membrane and accumulates in cytosol with a speed that depends on the concentration applied. Considering that the inhibition of SERCA pumps by TG is irreversible (Thastrup et al., 1990; Treiman et al., 1998), the number of inhibited SERCA pumps in the ER membrane likely increase with time following the TG accumulation in cytosol. This process can be accelerated with high TG concentrations, but it cannot be instantaneous and, therefore, it shapes the rising phase of TG-induced cytosolic Ca^2+^ transients. Previous experiments have shown, for instance, that the upstroke of Ca^2+^ transients generated by low TG concentrations of 0.1 and 0.5 µM is much slower than in those induced by 1 μM TG (Pick et al., 2021). As shown in Figure 3D, d[Ca^2+^]_cyt_/dt develops to a peak and decays rapidly to zero after TG application. This observation implies that the rising phase of the TG-induced Ca^2+^ transients followed a sigmoidal time course, which is the consequence of the cumulative inhibition of SERCA pumps by TG. When the ER Ca^2+^ depletion attained the maximal speed, the SERCA inhibition by TG is likely maximal and the Ca^2+^ leak from ER is fully unmasked. Hence, the second phase of the Ca^2+^ mobilisation reflects the Ca^2+^ leak from ER. By comparing the time courses of [Ca^2+^]_cyt_ and [Ca^2+^]_ER_ when SERCA inhibition is maximal, however, it became clear that the decay of the TG-induced cytosolic Ca^2+^ transients was much faster than the ER Ca^2+^ depletion (Figure 3C). In our hands, the TG-induced Ca^2+^ transients in HEK-293 and HEK-D1ER cells usually displayed such a fast decay in the absence of external Ca^2+^ (Figures 2A–4A). This observation may have several possible explanations. For instance, b[Ca^2+^]_cyt_ is generally in the range of approx. 100 nM. Such low Ca^2+^ levels in cytosol are maintained through the action of the PMCA and NCX (Barak and Parekh, 2020). Under these conditions, small amounts of Ca^2+^ leaked from ER may be sufficient to produce a prominent rise in the cytosolic Ca^2+^ levels. This suggestion is supported by the fact that there was less than 6% depletion of ER Ca^2+^ during this initial phase of the TG-induced Ca^2+^ mobilisation and yet the surge in [Ca^2+^]_cyt_ is produced by Ca^2+^ coming out from the ER (Figures 3C–F). On the other hand, concealed Ca^2+^ sources have been proposed also to explain large increases in cytosolic Ca^2+^ with minimal Ca^2+^ depletion in the ER/SR (Guerrero-Hernandez et al., 2010). This is not unexpected because the ER contains various proteins that bind Ca^2+^ such as calreticulin and BiP, which together buffer up to 75% of total ER Ca^2+^ (Prins and Michalak, 2011). In this view, the [Ca^2+^]_ER_ levels of HEK-293 cells are the result of a balance between Ca^2+^ leak from ER and mobilisation of concealed Ca^2+^ pools, i.e., the mobilisation of Ca^2+^ bound to luminal ER proteins. Thus, several mechanisms including Ca^2+^ buffering in ER lumen and in cytosol as well as Ca^2+^ clearance by the PMCA and NXC shape likely the second phase of the TG-induced Ca^2+^ mobilisation, when the ER Ca^2+^ is fully unmasked by TG.

In our modelling of Ca^2+^ transients, we fixed k_clear_ and used the Bateman equation to calculate k_leak_ values from fitting TG-induced Ca^2+^ transients that were obtained in the PURO experiments with HEK-D1ER cells. As a result, we obtained k_leak_ values that were approx. two times higher than those of k_depl_ (Table 1; Figures 4D, G, J; Figures 6A, C, E). This implies that the Ca^2+^ leak from the ER is much faster than the Ca^2+^ depletion in the ER. We found that k_leak_ was approx. one-half of k_clear_ under control conditions (Table 1). In order to understand the rules of cytosolic Ca^2+^ transients, next we broke the Bateman equation down into single terms (Figures 6B, D, F). Following the principle that the shortest rate constant of the Bateman equation determines the time course of the decay (see Garrett, 1994), our analysis showed that k_leak_ dominated in the decay of cytosolic Ca^2+^ transients. Accordingly, the TG-induced Ca^2+^ transient became shorter when k_leak_ was enhanced by the treatment with PURO, providing the rationale for the rule that the shortening of cytosolic Ca^2+^ transients is the best indicator for the enhancement of ER Ca^2+^ leak (Gamayun et al., 2019; Bhadra et al., 2021). In contrast, the amplitude of the cytosolic Ca^2+^ transients can increase or decrease in cells with enhanced ER Ca^2+^ leak, depending on the amount of depletion that produces the Ca^2+^ leak (Van Coppenolle et al., 2004; Lang et al., 2011b; Al-Mawla et al., 2020). Hence, the question arises on how much Ca^2+^ leak is required to modify cytosolic Ca^2+^ transients. Using Eqs. 7, 8 and the data presented in Table 1, we estimated the flux of Ca^2+^ ions out of the ER through Ca^2+^ leak channels (J_leak_). Under control conditions, for instance, a k_leak_ in the range of 14 e−3 to 16 e−3 s^−1^ will generate a Ca^2+^ leak of 5–6 µM⋅s^−1^ when the ER Ca^2+^ levels are around 370 µM. An enhancement of k_leak_ to 21 e−3 s^−1^ will increase the Ca^2+^ leak to about 8 µM⋅s^−1^ when the ER Ca^2+^ levels remain constant. This implies that at least a 33% increase of the Ca^2+^ leak is required to modify the TG-induced Ca^2+^ transients in a way that the duration becomes shorter and the amplitude larger, as shown in HEK-D1ER cells exposed to PURO (Figure 4A). These rough estimates of the Ca^2+^ leak in HEK-D1ER cells resemble the levels of the Ca^2+^ leak mediated by ryanodine receptors in cardiac muscle (Bers, 2014). When compared to other cell types, the Ca^2+^ leak of HEK-293 cells appears to be similar to those of professional secretory cells (Pick et al., 2021).

In conclusion, our quantitative data on parameters of the Ca^2+^ dynamics of HEK-293 and HEK-D1ER cells align with published data that has been obtained in Ca^2+^ imaging studies with various cell types. A one-compartment model satisfactorily explained the basic features of TG-induced Ca^2+^ transients and supported the rule that shortening of the TG-induced Ca^2+^ transients accompanied by an increased amplitude likely reflect the enhancement of ER Ca^2+^ leak (Figure 7).

## Data Availability

The original contributions presented in the study are included in the article, further inquiries can be directed to the corresponding authors.
